# Palm Sunday in central Mexico: among sellers, palms and syncretism

**DOI:** 10.1186/s13002-023-00587-3

**Published:** 2023-06-03

**Authors:** Jocelyn M. Briseño-Tellez, María Teresa Pulido Silva, Karen Bautista, Amairani García Mera, Omar Larios-Lozano, Berenice Nathaly López Gutiérrez, Yazmín Alejandra López López, Yesenia Mendoza Cruz, René Monzalvo, Daniela Ortega-Meza, Edith Carmina Sánchez Trejo, Zeltzin K. Zepeda-Hernández

**Affiliations:** 1 Centro de Investigaciones Biológicas, Universidad Autónoma del Estado de Hidalgo, Cd. Universitaria, Carr. Pachuca-Tulancingo, Km 4.5 s/n, C.P. 42184 Pachuca, Hidalgo Mexico; 2 Universidad Tecnológica del Valle del Mezquital, Carretera Ixmiquilpan-Capula, Km 4, Col. El Nith, C.P. 42300 Ixmiquilpan, Hidalgo Mexico

**Keywords:** Biocultural diversity, Commerce of palm leaves, Emic, Ethnobotany, Hidalgo, Markets, Non-timber forest products, Regional scale, Religious tradition

## Abstract

**Background:**

Domingo de Ramos, or Palm Sunday, is a traditional Christian religious event where devotees use *ramos*, which are bouquets currently elaborated from palm leaves and other natural elements. In various countries, it is assumed this use of biodiversity leads to the depletion of the species involved. However, other important aspects must be considered, including the role of the people who produce and sell these *ramos*, the associated symbolism that has been overlooked, as well as commercial aspects that have barely been documented. This ethnobotanical study evaluates the regional-scale cultural, biological and socioeconomic aspects associated with Domingo de Ramos in central Mexico from an emic perspective.

**Methods:**

Ethnographic and commercial information was obtained through interviews with *ramos* sellers in 28 municipalities in the state of Hidalgo, Mexico. We specifically sought sociodemographic data regarding the interviewees, as well as information pertaining to the *ramos* themselves and the palms. These aspects were explored with all of the sellers. The free list method was used to describe the uses and key elements associated with the *ramos*.

**Results:**

Although the *ramos* are used for religious purposes, they have eight different uses in the daily life of the sellers, the main one being “protection.” They serve to protect families, crops and animals, as well as against several diseases. Likewise, they are considered valuable for diminishing strong storms. This belief in the protection conferred by the *ramos* preserves pre-Hispanic concepts and is combined with their use in blessing corresponding to Western beliefs. *Ramos* are made from 35 introduced and native plant species and comprise a base (made of palm, wheat or sotol), a “reliquia” (palm, rosemary, chamomile and laurel) and natural or artificial flowers. The *ramos* sellers are mostly adult women of indigenous origin and heads of family.

**Conclusions:**

This study of Domingo de Ramos, carried out at a regional scale, highlights a syncretism that is reflected in both the symbolic importance of *ramos* palm and in the species used, as well as socioeconomic aspects that had not previously been identified in the study area and reflect the occurrence of complex relationships in non-timber forest products that remain little addressed.

**Supplementary Information:**

The online version contains supplementary material available at 10.1186/s13002-023-00587-3.

## Background

The practice of various religious festivals and ceremonies has been an aspect crucial to the preservation of the intangible value of nature, representing a millenary connection between biological and cultural diversity that conveys a vast biocultural abundance [1, 2]. *Domingo de Ramos* (Palm Sunday) is a religious tradition that marks the beginning of the Christian Holy Week. Research into this celebration has focused on the characterization of the biological species utilized [3, 4], pursuit of usage regulation [5, 6], description of aspects related to population status and trade [7, 8], and documentation of anthropological aspects [9]. However, the role of the people who elaborate and sell the *ramos* (Spanish for bouquet) on that day, and the associated symbolism has been overlooked. From an *etic* perspective, which is the researchers’ interpretation of different phenomena [10], it is understood that the *ramos* fulfill an ornamental function and that their only use pertains to Catholic celebrations. From an *emic* perspective, that is to say, the symbolism that the *ramos* could represent for those who use and trade them, the practice remains little understood. The present study, conducted in the central region of Mexico, focuses on the composition and symbolic meanings of the *ramos* from the sellers’ perspective, as well as characterizing their economic status and assessing the financial revenue generated by the trade in these *ramos*.

Domingo de Ramos is celebrated by the main Christian denominations (orthodox traditions, Catholicism, Lutheranism, Methodism, Anglicanism, the Moravian and Reformed churches). The devotees hold liturgy, in which they use palms (or olive branches) to commemorate the arrival of Jesus Christ to Jerusalem. Since the time of the ancient Greeks, palms have represented fertility and victory, and the Christian faith has adapted and associated them with sacred symbols; the palm represents heaven and its image has been linked to Christ and Mary [11, 12]. Mexico was colonized by catholic Spaniards who prioritized the catechization of indigenous people [13]. In Abya Yala (nowadays called America), there already existed prospering civilizations and cultures. Abya Yala has been inhabited by humans since 33,000 years ago [14], developing complex social and cultural relationships. The clash of two different worlds during the conquest caused a complex blend in many dimensions of life, including rituals. In Mesoamérica, Broda [15] proposes that current Catholic festivities are held around the time of the celebration of pre-Christian agricultural ceremonies. The blessing of seeds or Atlcahualo is known nowadays as Candlemas Day. Great Vigil or Huey Tozoztli is Day of the Cross. The Great Feast of the Lords or Huey Tecuilhuitl is the Assumption Day. Day of the Dead or Mijkailjuitl is All Saints’ Day [15]. Nonetheless, we do not have certainty if the study of processes that lead to syncretism during Holy Week has been widely documented, but see [16].

*Ramos* are currently elaborated in various countries using leaves from palm trees and some other elements. They are then blessed and taken to homes, where they remain for a year until the cycle restarts. These rituals create a clear demand for palms, which in many countries come from species inhabiting natural ecosystems; e.g., *Ceroxylon echinulatum* in Ecuador [7, 17], *Ceroxylon pityrophyllum* in Bolivia [8], *Attalea cohune* in Central America [18], *Attalea phalerata* in Peru [19], and *Acrocomia aculeata* in Honduras [20]. On other occasions, the palms come from plantations, some of which have been described as eco-friendly. This is the case in the USA and Canada, where an estimated number of 30 million palm leaves are utilized each year [21].

This ceremony generates 500,000 USD per year in Ecuador, with the commerce of palm leaves of *C. echinulatum* [7]. These authors indicate that its use has no detrimental environmental effects, and in fact has a positive impact on local economies [6, 7]. On the topic of commerce, two kinds of productive chains have been described for Domingo de Ramos: short chains and long chains [8]. Short chains maintain a few links (gatherer, weaver and seller), while long chains involve other intermediaries and gathering centers.

In Mexico, these commercial aspects have barely been documented, even though there is a considerable demand for orchids (e.g., *Prosthechea karwinskii*), Mexican laurel (*Litsea glaucescens*), bromeliads (e.g., *Tillandsia punctulata*) and cycads which are placed on the facades of churches and used to weave *ramos* [3, 4, 22–25]. However, inquiries regarding the palms used during the holiday only specify the species of plants, such as in the case of *Chamaedorea* spp. [26–28]*, Sabal mexicana* [29] and *Brahea dulcis* [3]. In other parts of the world, such as Europe, the flora is also widely used as a symbolic element in multiple festivities [30, 31].

In the past, ethnobotanical research in Mexico has usually been done in a few specific towns; however, wider-scale studies still need to be made available. Ethnobotanical studies are normally executed at a local scale, possibly due to the complexity of some of the studied phenomena, which justifies a focused, deep-dive approach. Other explanations could include limited research budgets, the limited size of available research teams, or simply the perceived lack of a need to answer the questions posed in the study. In contrast, regional studies are rare, but see [32, 33], although the number has tended to increase with time. Regional ethnobotanical studies could shed light on either the irregularity or the ubiquity of the phenomenon, and improve our understanding of processes that cannot be observed at a local scale [32].

The present ethnobotanical study evaluates the regional-scale biological, socioeconomic and cultural aspects associated with Domingo de Ramos in central Mexico. The objectives of the study were: (1) to elucidate, from an *emic* perspective, the composition and the symbolic meanings of the palms in central Mexico; (2) to document the sales volume of palms and their productive chain, as well as to characterize the socioeconomic status of the people conducting their commerce. The goal is to provide a panorama that integrates not only biological aspects, but also socioeconomic and cultural features regarding Domingo de Ramos, at a regional scale. Based on the previous aims, our hypotheses are: (1) since Domingo de Ramos is a Catholic tradition, it is expected that the symbolism behind the *ramos* will be associated with this particular religion; (2) considering the numerous natural palms distributed in the study area [34–36], it is expected that palms will be gathered from these sites and that the productive chains will be consequently short.

## Methods

### Study area

**Fig. 1:**
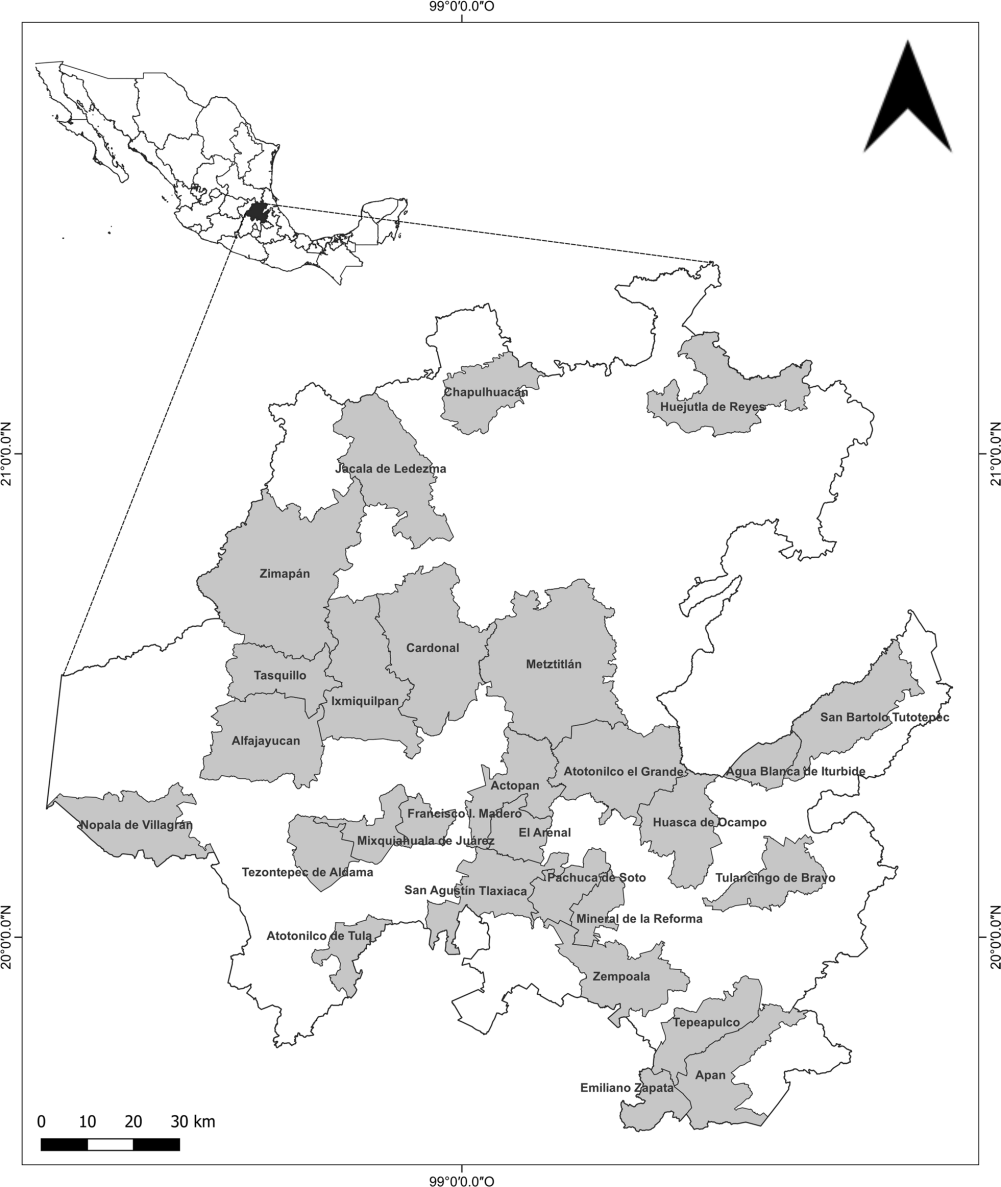
Municipalities in the state of Hidalgo where interviews were conducted during Domingo de Ramos, 2022

Twenty-eight municipalities were studied in central Mexico in the state of Hidalgo (Fig. 1). Hidalgo, which represents 1.1% of the national territory, is one of the states with the greatest biocultural diversity in Mexico, favored by its cultural, orographic and climatic complexity [37]. These territories comprise ten geo-cultural regions grouped by physiographic and cultural characteristics [38].

Three physiographic provinces converge in Hidalgo: the Transversal Volcanic System, Sierra Madre Oriental mountain chain and Gulf of Mexico Coastal Plain [39]. The warm and semi-warm climate zones of the Coastal Plain have average annual temperatures of 18 and 26 °C and precipitation of 1200 mm. In the temperate climate zone of the Sierra Madre Oriental and the Transversal Volcanic System, the average annual temperature ranges between 12 and 18 °C, with precipitation of 600–1500 mm. The dry and semidry climates of the Sierra Madre Oriental and the Transversal Volcanic System have average annual temperatures of between 12 and 22 °C, and precipitation of 300 and 600 mm [37, 40]. The anthropic area (agriculture, urban use and induced pasture) represents 53% of the state’s surface, followed by various types of native vegetation, dominated by scrub (13.5%), oak forest (8.3%), mountain forest (7.5%), coniferous forest (6.1%), and other coverages [41].

Hidalgo is inhabited by about 3 million people. The most populated studied municipalities are the state capital of Pachuca de Soto (314,331 inhabitants), Mineral de la Reforma (202,749), and Tulancingo de Bravo (168,369) [42]. The state has a 10 [42] to 33% [43] indigenous population. The predominant ethnic groups in the territory of Hidalgo are: Hñähñu in the Mezquital Valley, Nahua in the Huasteca Hidalguense, and, to a lesser extent, Tepehuas [42].

### Study design

Twenty-eight municipalities were selected, which cover eight out of ten geo-cultural regions, to get a broad overview of the diversity of plants required and their symbolic uses while allowing the documentation of their exclusive or non-common uses. In these areas, two types of semi-structured interviews were carried out. The first type of interview was applied to identify the gathering sites of the palm from which the *ramos* are made. The second type of interview was used to obtain descriptions of the vegetal elements contained in the *ramos*, their symbolism and the palm commercialization processes. To direct the sampling effort, only the municipal capitals were considered, since the most important churches and the main markets where the *ramos* are sold are located in these places.

### Interviews

Two semi-structured types of interviews were designed and applied following the recommendations of Bernard [44]. The first type was applied during the week prior to Domingo de Ramos (from April 3rd to 9th, 2022). A total of 40 interviews were conducted. While 28 sites were visited, only 12 municipalities had sellers. The number of interviews depended on the number of sellers that were found in each municipality. The first type of interview (Additional file 1) looked into these aspects: (1) data on the origin and costs of the palms; and (2) if the person collected the palm leaves, detailed questions were asked about the gathering and whether palm weaving is a tradition or if it is practiced solely as a means of generating income. In this first type of interview, we looked for sellers of palm because, in some cases, the leaves are sold without going through an artisanal process, instead sold wholesale or directly to the handcrafter, in other situations, *ramos* are sold during the days before Domingo de Ramos.

The second type of interview was done on Domingo de Ramos (April 10th, 2022). A total of 149 interviews were done in 28 municipalities. The number of interviews applied depends on the total number of vendors in each municipality (see Table 5). A previous recognition was carried out in order to count the family groups that sold *ramos*. When all the sellers were located, all the legal age ones that were in the place were interviewed, considering only one person per family group. In some cases, when the number of sellers was low, more than one adult person per family was interviewed. The second type of interview (Additional file 2) was structured around: (1) the sociodemographic data of the interviewees (age, place of origin, ethnic identity, etc.); (2) the *ramos* themselves (cost, plant species used, uses, symbolism); and (3) the palms (ways of obtaining them, origin, commercial agreements). These aspects were applied to all sellers of *ramos*. Furthermore, if the person was identified as a collector and/or weaver of leaves, specific questions relevant to these particular activities were asked. For palm collectors, the interview focused on the location of the sites gathered and the quantity of leaves gathered. Weavers were asked about what aspects make them feel proud of their work, what materials can be used as a substitute for palm, whether there is any governmental support for the handcrafter, among others.

For both types of interviews, all interviewed sellers gave their prior consent to participate (with verbal authorization). They were each given an introductory letter explaining the objectives and providing our contact number. Each interview lasted from 30 min to 1 h. Photographic evidence of the sales sites, churches, *ramos*, processions and sellers (with their prior consent) was taken. Seventy-seven *ramos* were purchased, which made it possible to determine a posteriori the botanical identity of the plants used. They were obtained in 20 municipalities from different sellers in order to have a sample of each design. All the *ramos* bought were chosen by their diversity of weaving style, materials and plants used. We sought to purchase the *ramos* with each of the individuals that were interviewed as long as the designs were not repeated.

A database was created in which the following information was registered for each *ramo*: a code to identify each seller, the place where the *ramo* was purchased, its sale price, among others (Additional file 3). These *ramos* were kept in the Ethnobiology Laboratory of the UAEH (Additional file 4) for subsequent deposition in the Ethnobotanical Collection of the UNAM.

### Analysis of the information

Based on the photographic evidence and the specimens acquired, a taxonomic list of the plants used in the elaboration of the *ramos* was generated. Botanical identification to species level was conducted based on their common names, taking into account the fact that most are widely known species. National and international risk categories according to NOM-059-SEMARNAT-2010 [45] and the IUCN [46] were indicated.

### Cultural domain on uses and composition of the *ramos*

The free list method was used to describe all the possible uses associated with the *ramos*, as disclosed by those who sell them, and the indispensable plant elements of which they are composed. This method allows an exploration of which elements make up a cultural domain [47]. The method consists of listing all of the items (uses, plant elements) mentioned by the interviewees and recording these in the order in which they were stated [44].

The items obtained in the free list were reclassified in order to group the responses considered related. This free list was analyzed using the Smith Index with the ANTHROPAC 4.0 program [48]. The Smith Index is calculated for each item as *S* = (Ʃ ((*L*-*R*_*j*_ + 1)/*L*))/*N*, where *L* is the length of the list, *R*_*j*_ is the average rank of each item _*j*_ in the list and *N* is the number of lists [49]. The importance of each item was determined according to both its frequency and order of mention. The index takes values between 0 and 1; the closer to 1 the value, the greater the importance of the item (uses, plant elements). In contrast, peripheral items present values close to zero.

### Palm obtaining chain, trade agreements and regional map of origin

The production chain refers to the set of agents (“economic agents”) -or their activities- involved in acquiring a final product. The chain involves everything from obtaining the resource (in industrial processes called raw material), to its transformation and final generation of the product for purchase by the final consumer [50]. On the scale of the 28 municipalities as a whole, an evaluation was conducted of the palm production chain for processing palm *ramos*. Since the palm is obtained from natural populations, the production chain of these non-timber forest products (NTFP) can best be understood as an obtaining chain. By palm obtaining chain, we refer to the agents and steps required to transform palm leaves cut from natural populations into a *ramo* for use in the Domingo de Ramos celebration.

For this, we followed the steps outlined by Bockel and Tallec [50]: (1) identification of activities and the flows that exist among them; (2) identification of agents in the chain; (3) design of a functional analysis table. In step 1, we focused on the agents directly related to the palm, while the suppliers of other elements (flowers, paper, prints) were not included. The elaboration of *ramos* is always handmade. Identification of agents (step 2) was conducted through observations and informal semi-structured interviews in markets and churches prior to Domingo de Ramos, following Bockel and Tallec [50], with special attention given to those agents who develop several roles simultaneously. Specific questions were elaborated for each role (therefore the second interview has sections exclusively for gatherers, weavers and sellers).

For each type of seller, it was determined from which type of agent the palm was obtained (e.g., gatherers, wholesaler). This was achieved by collecting information from the total number of interviewees in the 28 municipalities. The obtaining chain was represented in a graph. Trade agreements were tabulated and graphed. Prices were registered in Mexican currency (MXN) and US Dollars (USD) according to the Banxico exchange rate for March 23, 2023.

Finally, we traced where the palm leaves were brought to each municipality. This information was used to produce a map of the localities, tracing the origin of the palm leaves sold in those 28 municipalities and including their passage through wholesale and retail markets.

## Results

### Uses and meanings

**Fig. 2:**
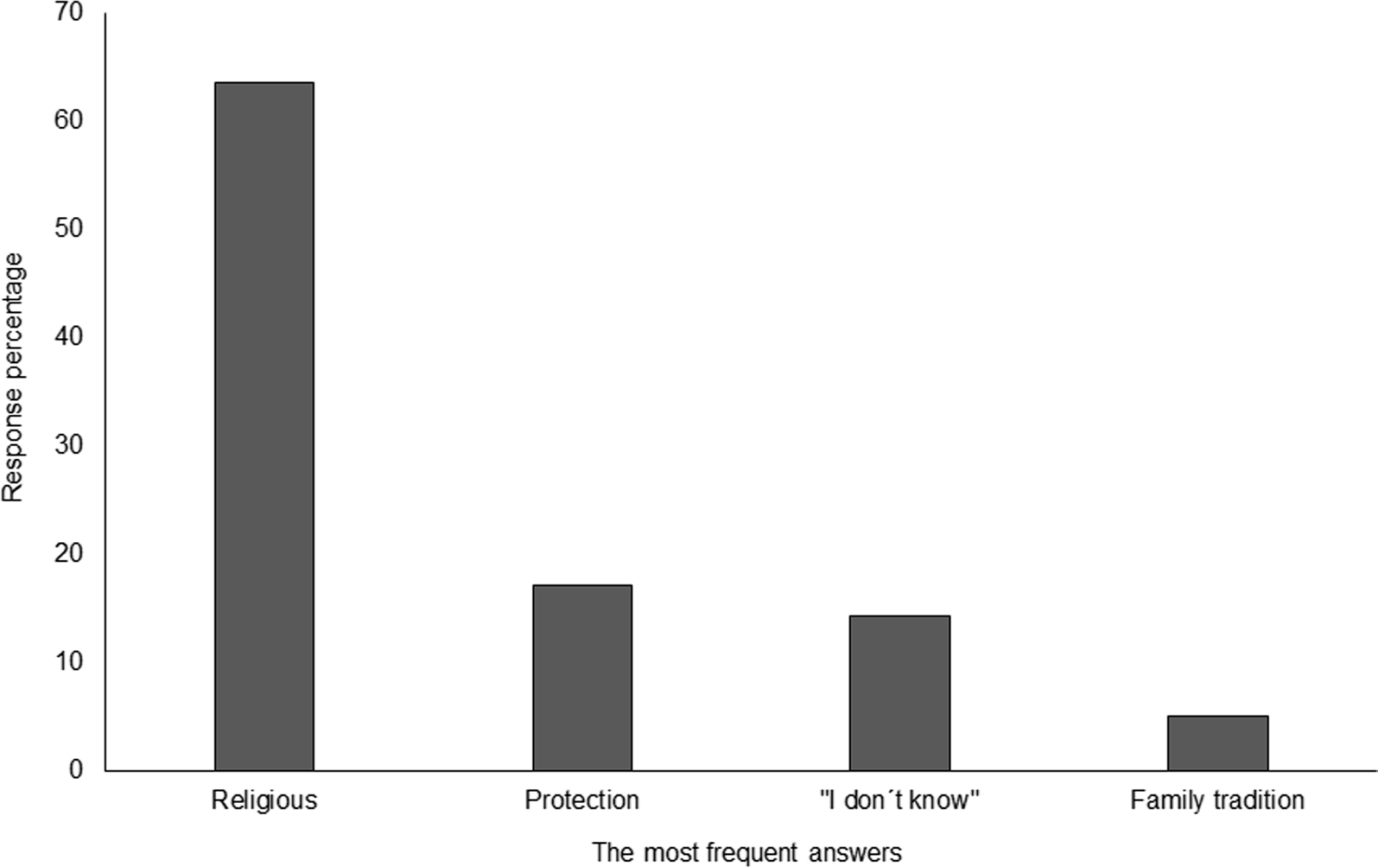
Significance of the *ramos* for the interviewed sellers

Considering the question, “what does the *ramo* represent?” The most common answer was its religious meaning (63.5%), but also protection, tradition, or some people simply “do not know” (Fig. 2). In contrast, the results demonstrate that, even though the palms are used for religious purposes, they have eight different uses, including protection (Smith index = 0.459), religious, blessing purposes, and others (Table 1). The protection use refers to the fact that the *ramos* are taken to the houses and placed behind the main entrance, where they serve to protect families (principally children), crops, and animals, as well as against several diseases (e.g., *el mal de ojo*, -the evil eye- and *el mal aire*, -the evil air-). However, above all, they are considered valuable for diminishing strong storms. In contrast, their religious use refers to “la entrada triunfal de Jesús a Jerusalén” (“Jesus Crist’s triumphant entry into Jerusalem”) and “el Miércoles de Ceniza” (“Ash Wednesday”). Their use for blessing purposes refers to the fact that the palms are taken to the church to be sprinkled with holy water.

**Table 1:** Frequency of mention of the uses of *ramos*

*Ramo* uses	Frequency (%) *n* = 149	Average range	Smith index
Protection	61.4	1.58	0.459
Religious	40.7	1.24	0.369
To bless	30.3	1.43	0.248
“Everything is used”	13.1	1.79	0.093
Decoration	8.3	1.58	0.062
Family tradition	4.8	1.86	0.036
“I do not know”	3.4	1	0.034
Burials	5.5	2.63	0.026
Trade sale	1.4	1	0.014

The least mentioned uses correspond to the categories “everything is used,” followed by decoration, family tradition, burials and sale. “Everything is used” reflects an ecological importance that must be highlighted. Some plants and flowers of the *ramo* are used as a spice or for medicinal purposes, while the “unblessed palms” are utilized as fodder for animals or they can be unwoven. The *ramo* can be boiled and dried and then used to make “petates” (mat), “aventadores” (winnowers), hats, kitchen brushes, and brooms, thus beginning a new useful life as a daily use item. The use of the *ramo* as decoration was assigned in this category since people commented that they used them as “adorno en la casa, después de la misa” (“decoration in the house, after Catholic Mass”) (Male, 59 years old). “Family tradition,” refers to the *ramo*, representing the activity of weaving every year, which is passed down between generations: “simboliza herencia familiar, nuestro trabajo” (“Our work symbolizes family inheritance”) (Female, 57 years old), “sé hacerlo desde niña, mi papá se dedicó a esto siempre y yo después aprendí” (“I know how to carry it out since I was a child; my father always dedicated himself to this activity, and subsequently I learned it”) (Female, 28 years old).

Interestingly, the use that presented the least importance for sellers was that of sale. At burials, the palm is used for the ceremony called “Levanta Cruz” (Raising the Holy Cross), in which the cross (made up principally of lime), placed during burial prayers held around the deceased, is collected with the blessed palm. Furthermore, 33 crosses are made with the *ramo* of blessed palms placed around the deceased. At the end of Domingo de Ramos, any *ramos* that are not sold are given to different family members and friends, donated to the church, or, as described above, reused to make other palm objects.

In addition to the utility of *ramos*, they are significant to the sellers. This asseveration was clear when asking the following question: “what makes you proud of your work with the palm?” The responses evidence that this activity is significant to the sellers because of the family heritage it represents and because palm weaving is a skill not possessed by everyone. One of the sellers interviewed reported, “es un orgullo saber tejer mientras que otros no saben cómo trabajar las palmas, yo creo que es un don que Dios nos ha dado” (“It is a pride to know how to weave when others do not know how to work the palms, I am convinced it is a gift God has given us”) (Female, 30 years old). Another interviewee said, “lo que más orgullosa me hace sentir es que conservo la tradición familiar, religiosa y al mismo tiempo es mi trabajo” (“what makes me feel the proudest is that I preserve the family and religious tradition, and at the same time it is my job”) (Female, 51 years old). Although the work carried out by the sellers is significant for them, the activity is little appreciated by those who demand the ramos for sale: “lo hago con cariño (el tejido de ramos), pero la gente lo regatea mucho y hace menos mi trabajo” (“I do this -activity of weaving ramos- with love, but people haggle a lot and demerit my work”) (Female, 22 years old). However, despite these facts, many sellers enjoy this activity year after year: “nada me molesta, me siento feliz con mi trabajo y con lo que me enseñaron” (“Nothing bothers me, I feel happy with my job and what I have been taught”) (Female, 42 years old).

### Composition

**Fig. 3:**
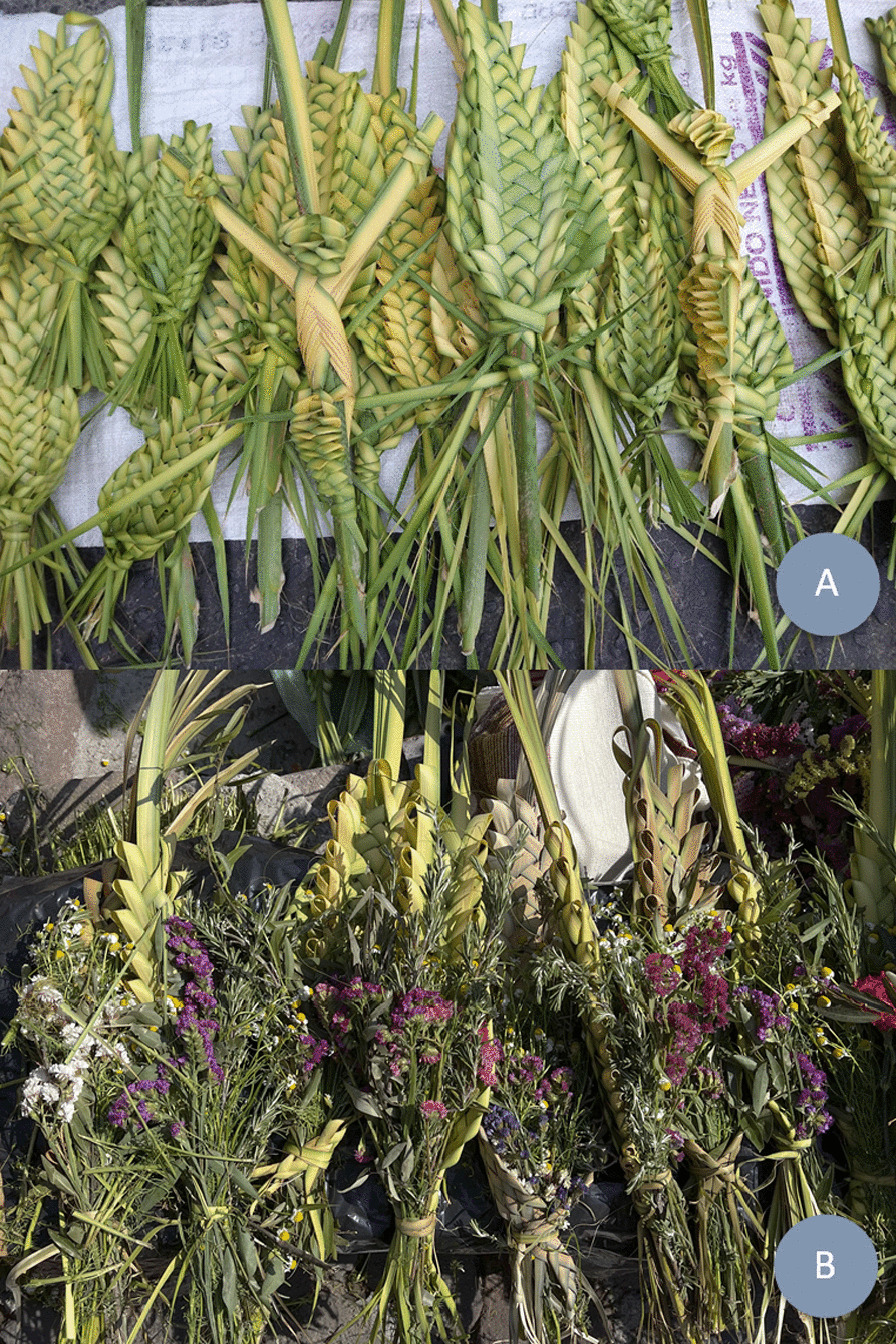
Composition and different forms of representation of the *ramos*: **A** without “reliquias” and in forms representing “Jesus Christ” and the “Virgin Mary,” Tulancingo, Hidalgo, April 10th, 2022. **B** accompanied by “reliquias” and natural flower (Sea lavender), Tepeapulco, Hidalgo, April 10th, 2022. Photo credit for **A**: Gutierrez-Arroyo N. M.; **B**: Ortega O

*Ramos* are made from 35 plant species, where the majority (24) are introduced species and, to a lesser extent (11) native species (Table 2). Generally, each *ramo* is made up of a base (made of palm, wheat, sotol), a “reliquia” (“relic”), and, in some cases, flowers (Table 2). Some interviewees recognize the “*reliquia*” as the palm and bouquet of rosemary, chamomile, and laurel, while other aromatic herbs (e.g., mint) are not part of the “reliquia.” For some people today, and especially in the past, a *ramo* is a green palm or sotol leaf with no weaving or other plants. Typically, *ramos* are woven into forms representing Jesus Christ or the Virgin Mary, accompanied by glitter, ribbons, and images of saints (Fig. 3). Of the plants used, only laurel (*Litsea glaucescens*) is listed as an “endangered species (*P*)” according to NOM-059 [45].

**Table 2:** Plant species used in the elaboration of *ramos*

Composition	Family	Scientific name	Authority names	Common name	Voucher no	Origin	NOM-059	IUCN
“*reliquia*” (“relic”)	Arecaceae	1.* Brahea dulcis*	(Kunth) Mart	Apak palm, blue rock palm (Palma soyate)	LE 32 LE 74	N	–	LC
	Asparagaceae	2. *Dasylirion* spp.	Zucc	Sotol	LE 48 LE 60	N	–	–
	Arecaceae	3.* Sabal mexicana*	Mart	Palmetto (Palma apachite)	LE 66 LE 69	N	–	LC
	Poaceae	4. *Triticum* spp.	L	Wheat (Trigo)	LE 20 LE 25	I	–	–
	Asteraceae	5.* Chamaemelum nobile*	(L.) All	Chamomile (Manzanilla)	LE 18 LE 41	I	–	–
	Lauraceae	6.* Litsea glaucescens*	Kunth	Mexican laurel (Laurel)	LE 6 LE 35	N	P	LC
	Lamiaceae	7.* Rosmarinus officinalis*	L	Rosemary (Romero)	LE 12 LE 58	I	–	–
Natural flowers	Amaryllidaceae	8.* Agapanthus africanus*	(L.) Hoffmanns	Lily of the Nile (Agapanto)	LE 30	I	–	–
	Verbenaceae	9.* Aloysia citrodora*	Paláu	Lemon verbena (Cedrón)	–	I	–	–
	Asteraceae	10.* Artemisia ludoviciana*	Nutt	White sagebrush (Estafiate)	–	N	–	–
	Asteraceae	11.* Bellis perennis*	L	Daisy flower (Margarita)	–	I	–	–
	Nyctaginaceae	12. *Bougainvillea* spp.	Comm. ex Juss	Paperflower (Bugambilia, camelina)	LE 2 LE 4	I	–	–
	Asteraceae	13. *Chrysanthemum* spp.	L	Chrysanthemum (Crisantemo)	LE 27 LE 29	I	–	–
	Asteraceae	14. *Conyza sophiifolia*	Kunth	Leafy marshtail (Simonillo)	–	N	–	–
	Caryophyllaceae	15. *Dianthus caryophyllus*	L	Carnation (Clavel)	LE 73	I	–	–
	Fabaceae	16. *Erythrina* spp.	L	Colorín or pemuche	–	N	–	–
	Euphorbiaceae	17. *Euphorbia milii*	Des Moul	Crown of thorns (Corona de Cristo)	–	I	–	–
	Onagraceae	18. *Fuchsia* spp.	L	Arete	–	I	–	–
	Geraniaceae	19. *Geranium* spp.	L	Cranesbills (Geranio, bola de fuego)	LE 29	I	–	–
	Asteraceae	20. *Gerbera*	L	Gerbera daisy (Gerbera)	–	I	–	–
	Caryophyllaceae	21. *Gypsophila paniculata*	L	Baby’s breath (Nube)	–	I	–	–
	Asteraceae	22. *Helianthus annuus*	L	Sunflower (Girasol)	–	N	–	LC
	Lamiaceae	23. *Jacaranda mimosifolia*	D. Don	Jacaranda	–	I	–	–
	Plumbaginaceae	24. *Limonium sinuatum*	(L.) Mill	Sea lavender (Estate, amortal, polar, flor de papel)	LE 13 LE 50	I	–	–
	Lamiaceae	25. *Mentha spicata*	L	Peppermint (Hierbabuena)	–	I	–	–
	Lamiaceae	26. *Ocimum basilicum*	L	Basil (Albahaca)	–	I	–	–
	Geraniaceae	27. *Pelargonium hortorum*	L.H. Bailey	Zonal geranium (Malvón)	–	I	–	–
	Ericaceae	28. *Rhododendron* spp.	L	Azalea	LE 43	I	–	–
	Rosaceae	29. *Rosa* spp.	L	Rose (Rosa)	LE 40	I	–	–
	Lamiaceae	30. *Ruta graveolens*	L	Rue (Ruda)	–	I	–	–
	Fabaceae	31. *Senna* spp.	Mill	Rosamaría	–	I	–	–
	Asteraceae	32. *Solidago velutina*	DC	Goldenrods (Solidago)	–	N	–	–
	Asteraceae	33. *Tagetes erecta*	L	Aztec marigold (Sempoalxochitl)	LE 43	N	–	–
	Liliaceae	34. *Tulipa* spp.	L	Tulip (Tulipán)	–	I	–	–
	Poaceae	35. *Zea mays*	L	Maize (Maíz)	–	N	–	LC

*NOM* Norma oficial Mexicana, *IUCN* International Union for conservation of nature, *I* Introduced, *N* Native, *P* Endangered species, *LC* Least concern, *LE* Laboratory of ethnobiology

A good *ramo* must include rosemary (Smith index of 0.628), chamomile (0.471), palm (0.403), natural flowers (0.338) and Mexican laurel (0.31) (Table 3). Likewise, the elements that *cannot be missing* from the *ramo* are rosemary (0.444), palm (0.279) and chamomile (0.231) (Table 4).

**Table 3:** Frequency of mention of plants and/or elements considered essential to produce a good *ramo*

Plants or elements	Frequency (%) *n* = 149	Average range	Smith index
Rosemary	84.4	1.97	0.628
Chamomile	74.1	2.48	0.471
Palm	49	1.75	0.403
Natural flowers	73.5	3.05	0.338
Mexican laurel	49	2.58	0.31
“*Reliquia*”	4.8	1.57	0.04
Images of saints	7.5	4.27	0.022
Artificial flowers	6.1	4.22	0.021
Rue	4.1	3.33	0.019
Lemon verbena	2	3	0.01
Wheat	1.4	2	0.01
Olive	1.4	2.5	0.009
Wooden stick	0.7	1	0.007
Sotol	0.7	2	0.005
Basil	0.7	3	0.004
Peppermint	0.7	3	0.004
White sagebrush	0.7	4	0.003
Hay	0.7	4	0.002

**Table 4:** Frequency of mention of the plants and/or elements that *cannot be missing* in the *ramos*

Elements	Frequency (%) n = 149	Average range	Smith Index
Rosemary	55.1	1.54	0.444
Palm	29.9	1.18	0.279
Chamomile	36.7	2.09	0.231
Mexican laurel	25.9	1.97	0.179
All are important	14.3	1	0.143
Natural flowers	19	2.11	0.121
“*Reliquia*”	2.7	2	0.016
Wheat	0.7	1	0.007
Olive	0.7	2	0.005
Artificial flowers	0.7	3	0.005
Rue	0.7	3	0.003

### Replacement of plant elements

**Fig. 4:**
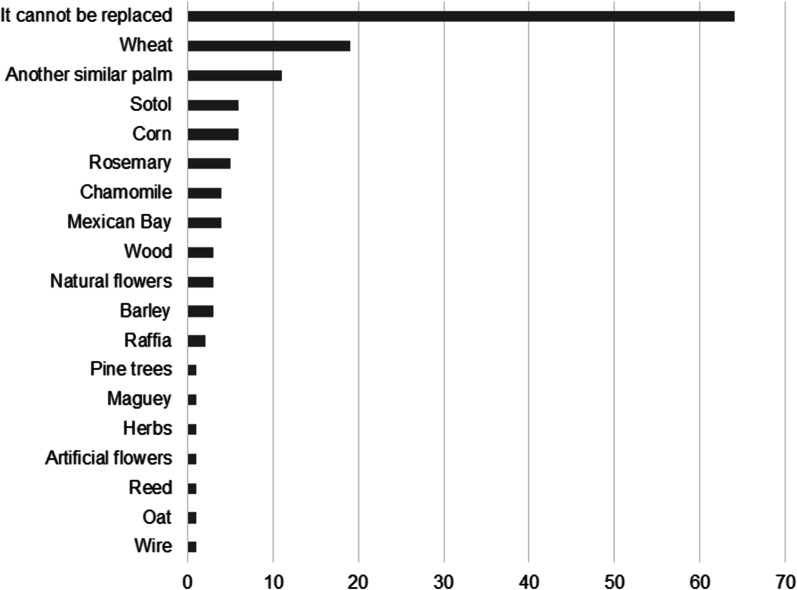
Substitute elements for the palm with which to elaborate the *ramos* utilized on the Domingo de Ramos celebration

The sellers report that some elements of the *ramos* are substituted with plastic or paper flowers due to the increased cost of natural flowers. *Ramos* made with sotol or common wheat are only found with glitter or images of saints. For the *ramos* made with palm, it was asked that, if for some reason the palm ran out, what other elements or plants could be used instead. The majority (64) of sellers replied that palm could not be replaced, while 19 believed it could be substituted with wheat, and 11 with another similar palm. The rest of the sellers considered it could be substituted with other elements (Fig. 4).

### Sociodemographic characterization of sellers

**Fig. 5:**
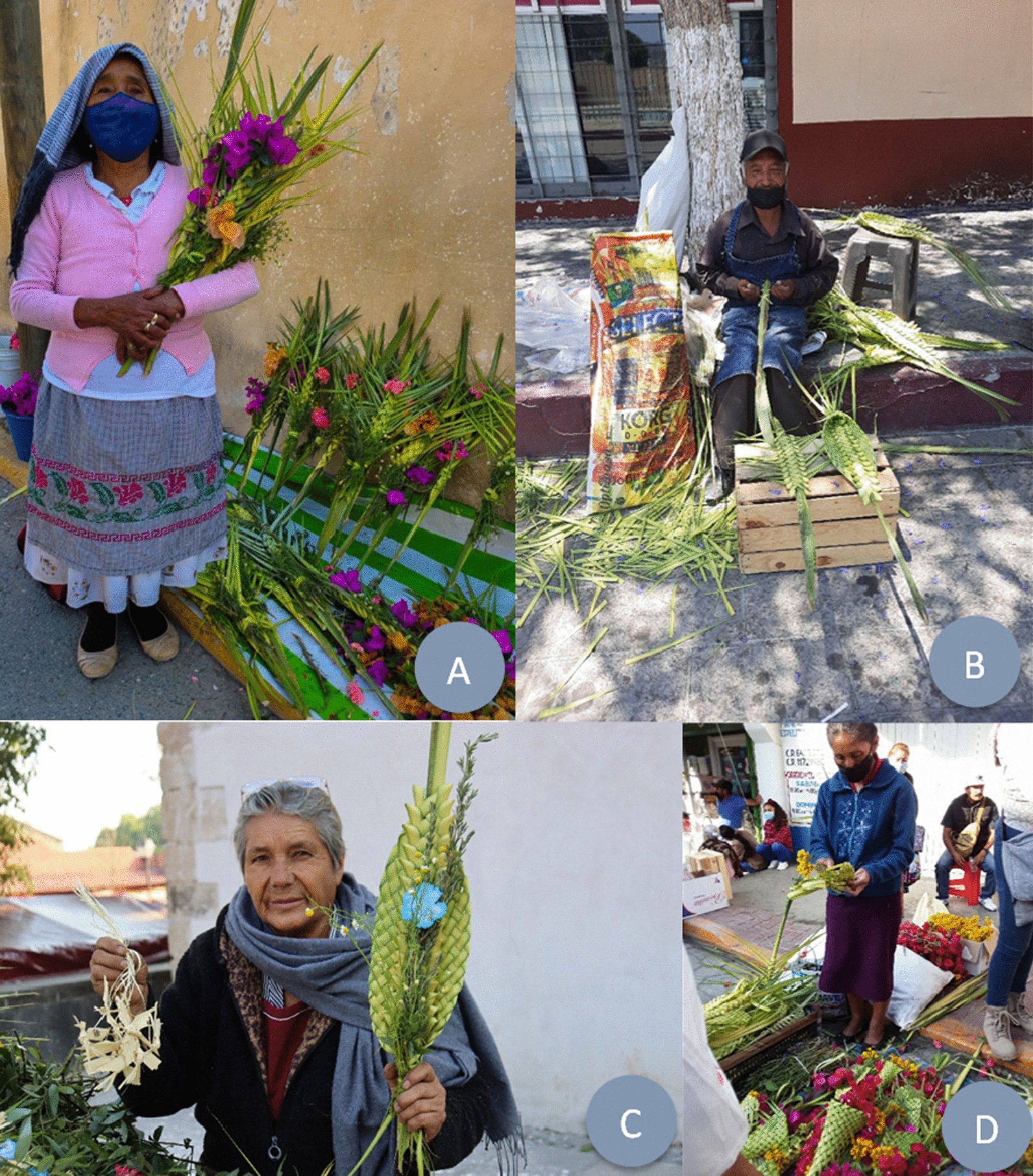
*Ramos* sellers in different municipalities of the State of Hidalgo on April 10th, 2022. **A** El Cardonal, **B** Actopan, **C** Huasca de Ocampo and **D** Jacala. The techniques used in palm weaving are varied and provide the opportunity to create delicate, meticulous and diverse works. The palm weaving is one of the tangible manifestations of intangible cultural heritage. Photo credit for **A**: Cardón- Jiménez, G.; **B**: Bautista, K.; **C**: Muñoz, A.; **D**: Pérez- González, L

**Fig. 6:**
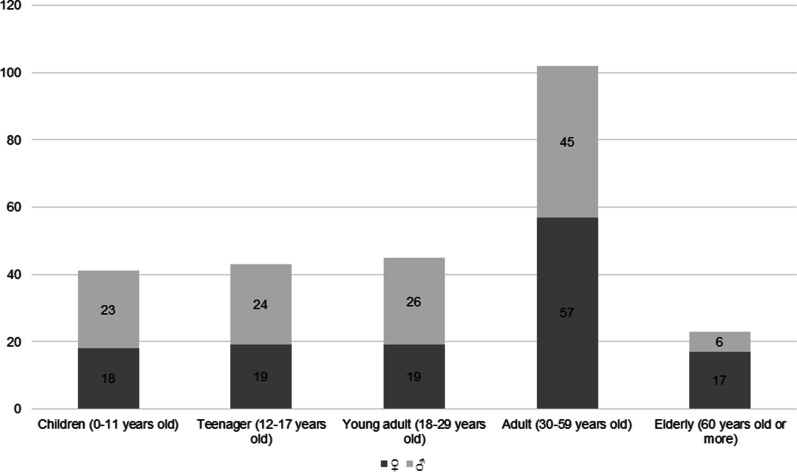
Number of *ramo* selling group members by age range and gender

The 71.8% interviewed on Domingo de Ramos were women, 54.4% recognized themselves as indigenous, and 64.4% were heads of the family. Commerce is their main economic activity (32.9%), followed by working at home (20.8%), others (17.4%), farming (14.8%), and handcraft sales (11.4%) (Table 5). The *ramo* sellers (Fig. 5) came from the states of Hidalgo (144), Mexico (19), San Luis Potosí (8), Puebla (8), Tlaxcala (4), Veracruz (3), and Querétaro (1). A total of 96% of the vendors work in groups, mainly comprising adults (30–59 years old) (40.5%) and, less frequently, older adults (60 years old or above) (Fig. 6). It is typical for the different group members to divide the activities during the day; some are dedicated to palm weaving, others to attaching the “*reliquias*” or the flowers that the *ramos* feature, while others are in charge of sales. Due to the challenging nature of the activities, cases in which the sellers work alone are infrequent.

**Table 5:** Sociodemographic information of sellers

		First type of interview		Second type of interview																		
		# Interviewees		# Estimated		# Interviewees		Identity	Spoken language				Main economic activity					Secondary activity	Sales strategies			
Study area	Total, population INEGI 2020	F	M	Sellers	Groups	F	M	Indígenous	Ñhañhú	Náhuatl	Others^+^	Householder	Commerce	Home	Others*	Farming	Handcrafts	Ramos sales	Strategy 1**	Strategy 2**	Strategy 3**	Strategy 4**
Actopan	61,002	1	1	14	8	5	3	0	0	0	0	7	4	0	0	4	0	0	0	0	0	8
Agua Blanca de Iturbide	10,313	1	0	11	5	4	1	1	0	1	0	1	3	2	0	0	0	3	2	0	1	2
Alfajayucan	19,162			7	6	6	0	6	3	0	0	5	0	3	0	1	2	2	0	0	2	4
Apan	46,681	–	–	30	4	3	5	0	0	0	0	3	4	1	3	0	0	4	1	0	3	4
Atotonilco de Tula	62,470	–	–	5	3	3	0	0	0	0	0	3	3	0	0	0	0	0	0	0	2	1
Atotonilco el Grande	30,135	4	0	58	15	7	2	8	3	0	0	8	0	5	2	1	1	5	6	0	0	3
Cardonal	19,431	1	3	4	4	3	1	4	4	0	0	3	0	2	1	0	1	1	1	0	0	3
Chapulhuacán	22,903			33	11	6	3	3	0	2	0	7	1	6	0	2	0	0	0	1	1	7
Emiliano Zapata	15,175	2	1	9	2	2	0	2	0	1	0	2	1	1	0	0	0	1	0	0	0	2
Huasca de Ocampo	17,607	–	–	2	2	2	0	1	0	0	0	1	1	1	0	0	0	1	0	0	1	1
Huejutla de Reyes	126,781	5	0	37	7	2	3	5	0	5	0	3	0	0	1	0	4	0	3	0	0	2
Ixmiquilpan	98,654	4	0	9	1	8	1	7	3	0	0	8	2	1	1	0	5	4	0	1	4	4
Jacala de Ledezma	12,290	–	–	14	8	6	3	1	0	1	1	5	6	0	2	0	1	4	4	5	0	0
Mineral de la Reforma	202,749	–	–	7	2	2	0	1	0	0	0	1	2	0	0	0	0	1	0	0	0	2
Mixquiahuala de Juárez	47,222	0	1	107	12	3	1	1	1	0	0	1	2	0	2	0	0	0	1	0	1	2
San Bartolo Tutotepec	17,699	–	–	2	2	1	1	2	0	1	0	2	1	0	1	0	0	0	0	0	0	2
Tasquillo	17,441	–	–	27	14	4	3	7	7	0	0	5	2	1	1	3	0	2	6	0	0	1
Tepeapulco	56,245	3	2	7	2	2	1	3	0	3	0	2	3	0	0	0	0	0	0	0	0	3
Tezontepec de Aldama	55,134	–	–	15	5	5	2	6	0	0	0	5	2	1	2	1	1	2	0	1	2	4
Zempoala	57,906	–	–	2	1	2	0	0	0	0	0	0	2	0	0	0	0	0	0	0	0	2
Francisco I. Madero	36,248	1	0	21	9	5	3	6	4	0	2	6	2	1	3	2	0	0	0	0	1	7
Tulancingo de Bravo	168,369	5	0	14	8	8	0	2	0	2	0	6	1	3	1	2	1	6	2	0	0	6
Pachuca de Soto	314,331	–	–	52	4	6	1	0	–	–	–	0	0	1	2	0	0	0	1	0	1	5
El Arenal	19,836	–	–	100	30	3	2	3	1	0	0	2	3	0	2	0	0	2	0	0	1	4
Metztitlán	20,962	–	–	54	5	4	5	7	6	0	0	7	1	2	0	6	0	4	8	0	1	0
Nopala de Villagrán	16,948	–	–	2	2	2	0	2	1	0	0	1	0	0	1	0	1	0	0	1	0	1
San Agustín Tlaxiaca	38,891	–	–	2	2	1	1	1	1	0	0	1	2	0	0	0	0	0	0	0	0	2
Zimapán	39,927	5	0	8	8	2	0	2	0	0	0	1	1	0	1	0	0	2	0	0	0	2
	Total	32	8	653	182	107	42	81	34	16	3	96	49	31	26	22	17	44	35	9	21	84
	%	80	20	–	–	71.8	28.2	54.4	22.8	10.7	2.0	64.4	32.9	20.8	17.4	14.8	11.4	29.5	23.5	6.0	14.1	56.4

*Builder, firework job, seamstress, student, teacher, public server

**Strategy 1: gather and weave the palm and sell the *ramos*; Strategy 2: same as above and additionally purchase palm leaves; Strategy 3: only sell *ramos*; Strategy 4: purchase palm leaves, weave and sell *ramo*

^+^English and Maya

### Palm leaf obtaining chain

We identify five stages of the chain, that include every step, from palm leaves acquisition to retail sale. Each stage has an essential function for the commercial chain; for example, palm leaf acquisition refers to the collection of leaves. This function is performed by the gatherers, which makes it possible for the palm leaves to be marketed to other agents as weavers, intermediaries and wholesalers. This functional analysis helps show the interaction between agents and their function since several activities can be conducted, occasionally, by the same person. Such is the case with sales. Seven classes of actors were recognized, including gatherers, intermediaries and sellers, and they may perform five activities (Table 6).

**Table 6:** Functional analyses

Stage of the chain	Function	Agent	Output
Palm leaves acquisition	Gather	Local gatherer	Palm leaves marketed to local weavers or local intermediaries
	Gather	Non-local gatherer	Palm leaves marketed to local intermediaries or wholesalers
Primary Marketing	Transportation and facilitating the gathering of leaves	Local intermediary	Transportation of leaves to a known sales site in local squares and markets Sale, usually in units of 100 leaves
	Transportation and facilitating the gathering of leaves	Wholesaler intermediary	Transportation of leaves to extensive non-local markets Sale, usually in units of 100 leaves
Craft transformation	Weave palm leaves Sale	Weaver–seller (Strategy 4; W, S)	Fabricating the *ramos* Sale of *ramos* outside churches
Marketing	Sale	Intermediary seller (Strategy 4; P, S)	Purchase the already woven palm leaves (*ramos*) Sale of *ramos* outside churches
Retail sale	Final sale	Seller	Sale of *ramos* to the final consumer

**Fig. 7:**
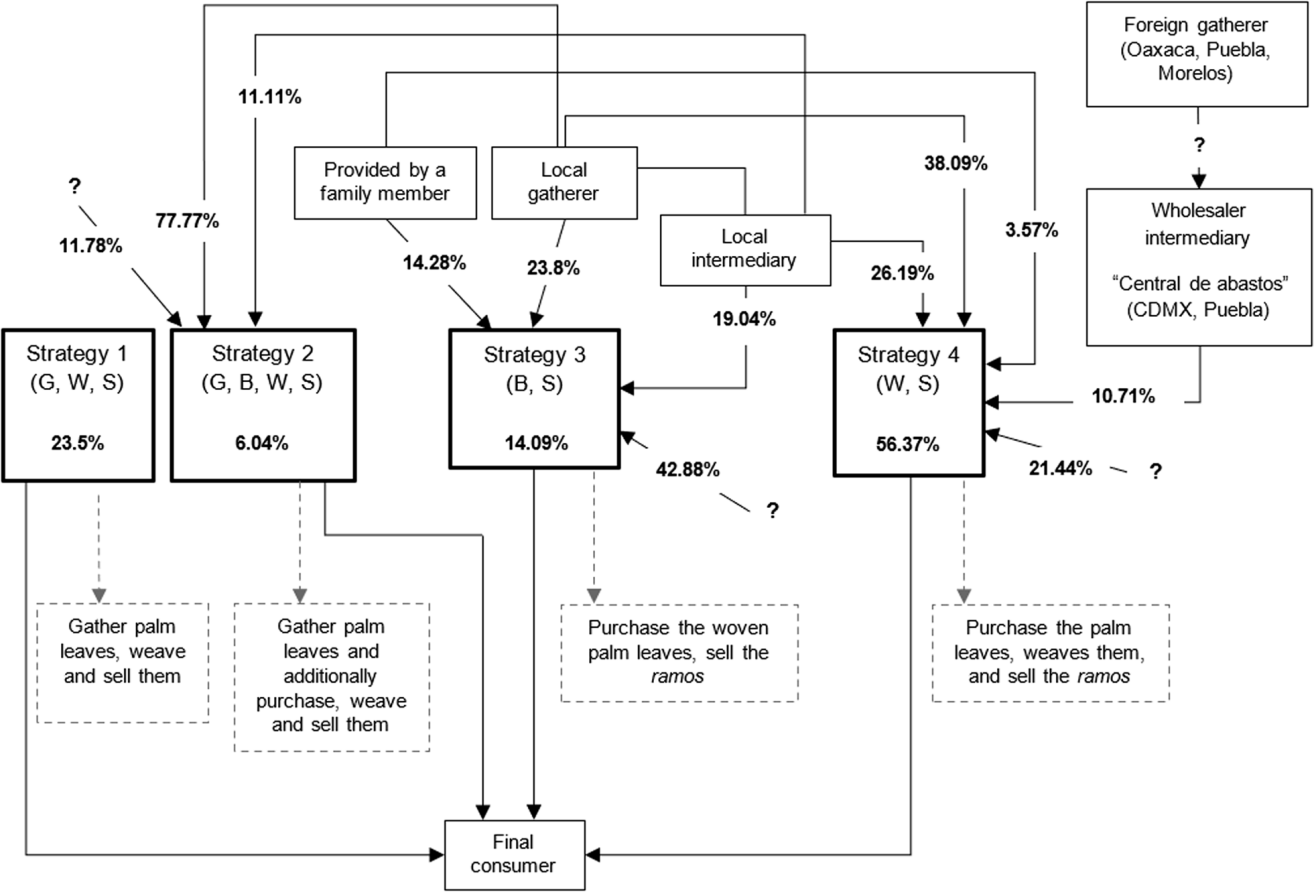
Palm leaf obtaining chain employed to elaborate the *ramos*. The four types of sellers are shown with boxes highlighted in black. Cases in which the interviewee did not know the role of the person from whom he/she obtains the palm or did not supply information are denoted with a question mark

**Fig. 8:**
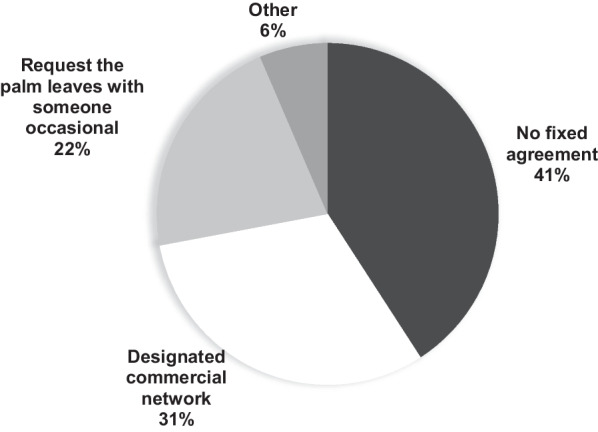
Commercial agreements for the purchase of palm leaves

Four types of sales strategies were found: (1) “Strategy 1 (G, W, S)”: gather and weave the palm and sell the *ramos*; (2) “Strategy 2 (G, P, W, S)”: same as above and additionally purchase palm leaves; (3) “Strategy 3 (P, S)”: only sell *ramos*; (4) “Strategy 4 (W, S)”: purchase palm leaves, weave and sell *ramos* (Fig. 7). The first stage in the chain is acquiring the palm leaves; this activity is conducted by gatherers (local or from outside the region) who choose the new leaves (closed). In the case of local gatherers, the cutting of leaves is accomplished eight to 15 days before the Domingo de Ramos celebration. For this assignment, the local gatherers say it is necessary to pay for a permit (approximately 500 MXN or USD 26.86) authorizing palm collection. The second stage is direct marketing, which is carried out mainly by intermediaries and consists of transporting the leaves to known sales sites in local plazas and markets. Leaves can be purchased in units of 100. The third stage is handcraft transformation: the weavers assemble the *ramos* (weaving the palm leaves, attaching the “*reliquia*” and flowers). A fourth stage, dubbed “marketing,” may occur when some people are intermediaries and only attach the “*reliquia*” and flowers. The final stage is that of the sale, which takes place outside or close to the churches where the *ramos* reach the final consumer (Fig. 7). Among the commercial agreements to purchase palm leaves, the most common is that there is no fixed agreement (41%); i.e., the consumers of leaves go to the known sales sites (plazas or markets) and buy according to existing demand. In other circumstances, there is an established commercial network (31%), or request the palm leaves with someone occasional (22%) (Fig. 8).

### Acquisition cost of palm leaf, sale of *ramos*, and expected earnings

**Fig. 9:**
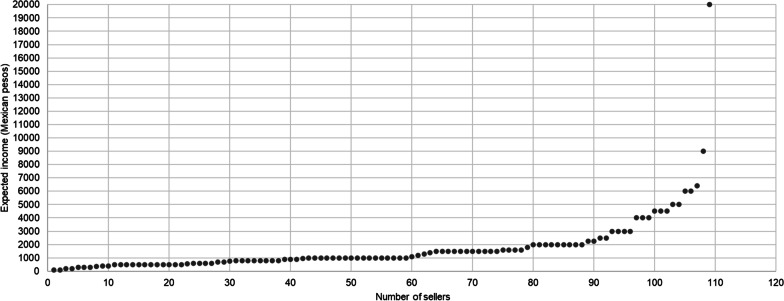
Income that the interviewed sellers expect to earn from the sale of *ramos*

Sale of *ramos* represents the secondary economic activity for 29.5% of the sellers interviewed (Table 5). According to that stated in the interviews, it was estimated that 19,078 *ramos* would be sold during the Domingo de Ramos event. The sellers consider multiple factors when assigning a monetary value to the *ramo*, including the size, the flowers they contain, the processing time, and/or the acquisition cost of the palm leaves. Each palm leaf can be bought for 2 MXN to 5 MXN (from 0.11 to 0.25 USD) or it can be purchased in bulk: 100 leaves commonly cost from 150 to 300 MXN (8.09–16.18 USD) in Hidalgo, and from 350 to 500 MXN (18.88–26.86 USD) in markets in Mexico City. *Ramos* vary in price from 10 to 40 MXN (0.54–2.16 USD), but they can usually be purchased for 25 MXN (USD 1.35). It was also shown that *ramos* (without other plants) can be purchased by the dozen, and their cost in this form ranged from 70 to 90 MXN (3.76–4.83 USD). All of the prices given above can vary depending on the particular commercial agreements (Fig. 8) established between people; as an interviewee explains: “el precio (de una hoja) normalmente está a $5 MXN, o sea, a 500 el ciento, pero por el acuerdo comercial que hice, el ciento me salió en $150 MXN ($1.50 MXN cada hoja)” (“the cost -of a palm leaf- is typically at 5 MXN (USD 0.27), that is, 500 MXN (USD 26.86) per one hundred leaves, but due to the commercial agreement I made, the one hundred came out at 150 MXN (USD 8.06) -or 1.50 MXN (USD 0.081) per palm leaf-”) (Male, 23 years old). The results suggest that more than 90% of the sellers (100) expect to obtain an income of less than 5000 MXN (USD 268.58). In contrast, the remaining 10% (< 10) anticipate earnings comparable to or greater than 5000 MXN (USD 268.58), with a maximum reported income of 20,000 MXN (USD 1074.32) (Fig. 9). This income is mainly used to pay for maintenance and food (85.9%), while 12.7% is destined for other purposes, such as saving, reinvestment of the money, or supporting local activities.

### Circulation of the palm leaves

**Fig. 10:**
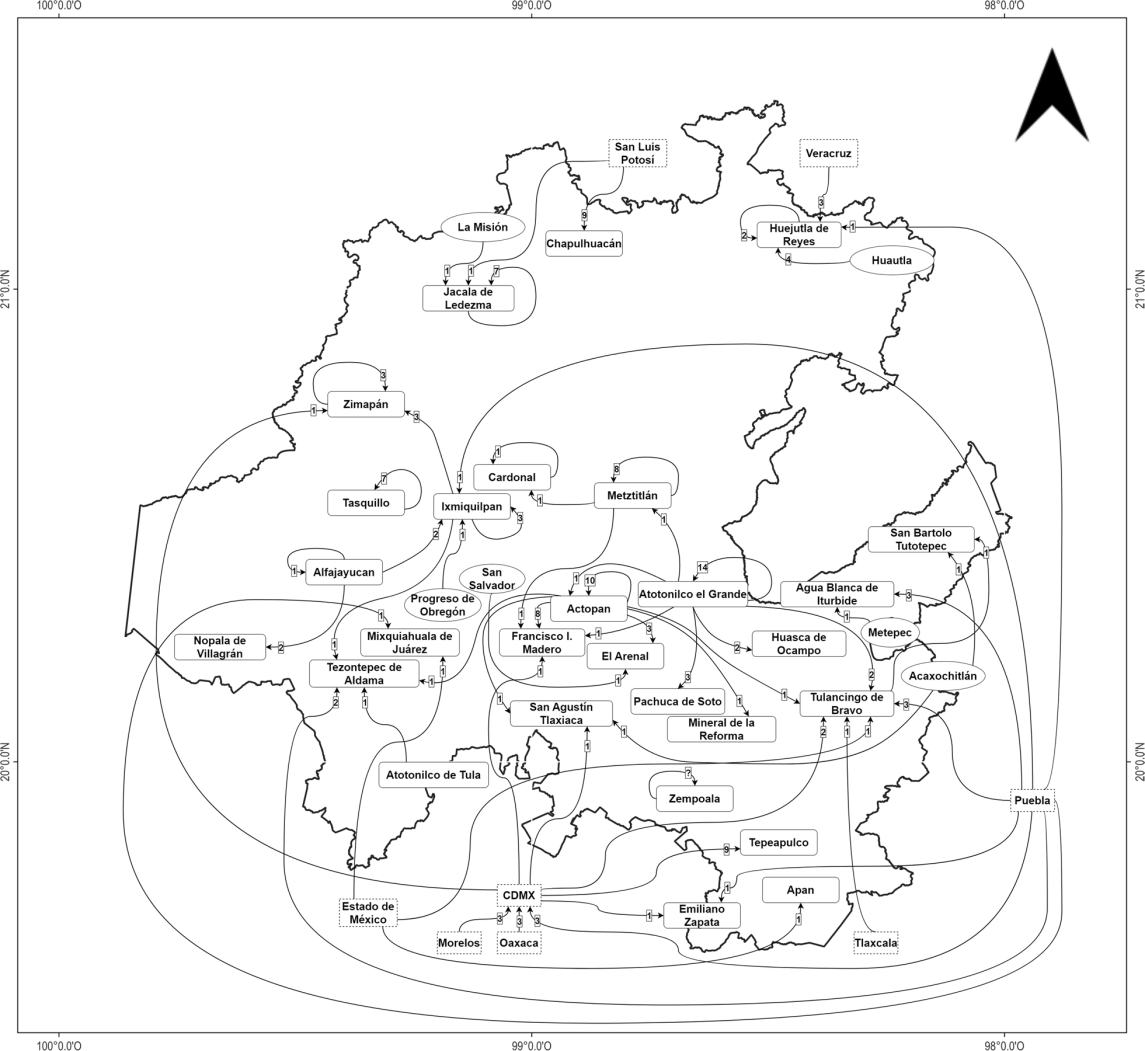
Circulation of the palm leaves sold on Domingo de Ramos 2022. The rectangles denote the locations where the interviews were conducted; the ovals show the municipalities from which the palm leaves come, but which were not considered in this study (Huautla, La Misión, Metepec, Progreso de Obregón and San Salvador). The arrows indicate the direction of movement between the source and the destination of the palm, while the numbers indicate the frequency with which each location was referred to as a collection site

Based on the interviews, information was obtained regarding both the natural populations and the palm source markets. In Hidalgo, the principal markets are in Huejutla (North), Tulancingo (East), and Ixmiquilpan (Center); however, there are other places from which palm leaves can be sourced. Actopan and Atotonilco el Grande are considered the leading sites for obtaining leaves. For the northern zone of Hidalgo, the palm comes predominantly from natural populations within the same localities as their final use, but also from San Luis Potosí and Veracruz. In the eastern part of the state, the palm can be acquired in markets and from the natural populations of Puebla and Tlaxcala. Toward the south, the palm leaves are obtained from large wholesale markets (“Centrales de abastos”) in Mexico City and Puebla. In these cases, the original source of the palm is almost certainly unknown. However, the sellers who move directly to the supply centers and have always procured goods from the same place, report that the palm comes from Oaxaca, Morelos, and Puebla (Fig. 10).

## Discussion

Domingo de Ramos, in central Mexico, is a syncretism between pre-Hispanic beliefs and Catholic religion, reflected both in the species used and in the associated symbolism. On the one hand, the plants used to make *ramos* are a mixture of native Mexican species and others introduced from Europe. On the other hand, the use of *ramos* has protective and medicinal elements and is not only used for what is referred to as Catholic ritual. This dialectical interaction between the two beliefs has been documented in certain religious festivities [2, 24, 51] and constitutes an aspect of identity in Mexico.

### Significance of the *ramos*: between pre-Hispanic protection and Catholic ritual

While the interviewees recognized the Catholic significance of the *ramos*, in the daily life of the sellers they have other uses. Their main use is as “protection,” a use in which pre-Hispanic beliefs are still preserved. For this reason, we kept “protection” and “blessing” as two distinct categories in the study. This belief in the protection conferred by the *ramos* preserves pre-Hispanic concepts and rituals, while their use in blessing corresponds to Western beliefs. The *emic* interpretation goes beyond the Catholic conception, thus rejecting our first working hypothesis.

Some research about the use of plants for religious festivities and their symbolic importance are also reported in Poland for Assumption Day (August 15). People make bouquets mainly of medicinal herbs on this festivity, which also have a protective and apotropaic use against evil influences. The bouquets are hung on walls, inside one’s house or on farming premises and are still used for smudging in protective magic against storms and thunder or may be laid under a deceased person’s head. In this research, *rozchodnik* or goldmoss stonecrop (*Sedum acre* L.) concerns protection from thunder; these beliefs concerning the powers of this plant are common throughout Europe and can be traced to Roman times [52].

We could consider many species of plants as living examples of history that continues until our days because they retain symbolic, ritual and medicinal uses that are very difficult to separate from each other. They have probably overlapped on many occasions since antiquity [53].

Uses of the *ramo* as a protector of people or crops have been described as paganism. Thus, maintenance of these beliefs has been seen as an illegitimate form of faith: “lo verdaderamente importante es participar en la procesión y no simplemente procurarse una palma o ramo de olivo; que estos no se conserven como si fueran amuletos, con un fin curativo o para mantener alejados a los malos espíritus y evitar así, en las casas y los campos, los daños que causan, lo cual podría ser una forma de superstición” (“what is truly important is to participate in the procession and not simply procure a palm or olive branch; these should not be kept as if they were amulets, for healing purposes or to ward away evil spirits and thus prevent the damage they cause in the homes and fields, which could be a form of superstition”) [54, 55]. Similarly, in Spain, for the same celebration, these protective uses of *ramos* have been interpreted as a link between popular religiosity, magic, and nature and have been described as “profane remedies” [56].

The term “reliquia” (“relic”) merits a special mention. Some interviewees recognize the “reliquia” as part of the *ramo*, referring to the rosemary, chamomile, laurel, and palm. These are used as spices or medicinal herbs. At the same time, in the study area, Guerrero [57] reports the use of “reliquia” in other contexts; e.g., to avoid miscarriage (using rosemary, fennel and marigold); for “limpias” (“healing rituals”); and to avoid children being hurt by witches (apotropaic use). The broad use of the term “reliquia” is relevant since, for the Catholic religion, it refers to parts of saints or objects that have been in contact with them, and attributes a sacred and miraculous significance [58].

*Brahea dulcis* palm, considered a “reliquia,” has a very important protective use. On the one hand, in funerary contexts, the deceased are protected with *huaraches* (sandals), crowns and crosses made of palm so that they can safely reach the afterlife. This is practiced among the Hñähñu, Nahuas and Ixcatecos in Hidalgo, Guerrero and Oaxaca [36, 59–61]. In addition, it is used to protect people (especially children) and homes (this study and [36]) and is widely used to calm storms. This probably reflects pre-Hispanic conceptions, as has been explored in anthropology through studies on lightning ritualists [62, 63]. These *emic* approximations, which result from the syncretic process, merit further study.

### Vegetal species

The species identified as essential to make a “good *ramo*” are of European origin: chamomile (*Chamaemelum nobile*), rosemary (*Rosmarinus officinalis*), rue (*Ruta graveolens*) and olive (*Olea europaea*). The latter three have been documented in Spain in relation to Domingo de Ramos, where rosemary is venerated for its relationship with the story of the Virgin, rue is considered protective due to its powerful smell and the olive tree symbolizes peace [56]. These introduced species in Mexico are used together with native plants such as the *Brahea dulcis* palm and the Mexican laurel (*Litsea glaucescens*) (its European equivalent *Laurus nobilis*) is a symbol of victory and eternal life accordance to [56], which reflects the syncretism.

In Europe, many plants are used during Palm Sunday, which are blessed and have an apotropaic use. In Poland, Kujawska et al. [64] carried out historical ethnobotanical research where nearly 250 studies of plants and their use in folk culture are documented, describing medicinal, magic, apotropaic, ritualistic, among other uses. Below we mention some of these plant species blessed during Palm Sunday in Poland; *Brzoza* or birch (*Betula verrucosa* Ehrh.), is believed to have healing and magical powers and is considered a lucky tree. *Cis* or yew (*Taxus baccata* L.), despite being a toxic plant, is used in *ramos*; likewise, sick cattle are incensed with it and it also protects persons from witches, misfortunes and all evil. *Leszczyna* or hazelnut (*Corylus avellana* L.) is considered magical and protective usages against lightning; crosses made with this plant are hung over doors and windows to prevent the access of an evil spirit. *Jałowiec* or juniper (*Juniperus communis* L.) is used in houses and livestock against witchcraft. *Jemioła* or mistletoe (*Viscum album* L.) once blessed, is believed to have power and strength against curses. *Jodła* or silver fir (*Abies alba* Mill.), although considered unlucky tree and not planted next to houses, is added to the blessed palm on Palm Sunday. *Kłokoczka* or bladdernut (*Staphylea pinnata* L.) is used to make *ramos*; local people also attributed great magical power to it. *Kocanki* or sandy everlasting (*Helichrysum arenarium* L.) is tied in *ramos* which protect against lightning when hung on the walls. Also, in Ukraine, other studies reported that *Tagetes patula* L. has a ritual use that is blessed on Assumption Day and Palm Sunday [65], and branches of willow (*Salix* spp.) on Palm Sunday; it protects against evil and aids in beating out the devil [66].

### Substitution phenomena

*Ramos* were originally made with natural plant elements that can be returned to the soil and can easily be degraded. Since the price of natural flowers increases the purchase costs, cheaper artificial flowers are being used to replace them. This substitution leads to changes in significance, since each element of the *ramo* has a very deep symbolism, although some interviewees included artificial flowers as elements that cannot be missing in a “good *ramo*.” The use of plastic elements occurs in different religious festivals, where some authors state that this practice detracts from the purity and cultural value of the celebrations [67, 68].

### Historical changes

An interesting aspect that needs to be addressed in future research is the changes in the composition and symbolism of the *ramos* over time. In some European countries, it has been observed that one of the most important reasons for those changes on *ramos* for Assumption Day is the transformations of the vegetation and lifestyle in rural areas [69]. This kind of research could give a glimpse into transformations in the landscape and their influence on traditions. While the present work contributes to describing in detail the contemporary uses and meanings, the historical changes in the uses of the *ramo* in central Mexico must be considered.

### Are the ramos associated with domingo de *Ramos* “eco-friendly”?

Palm leaves gathered from plantations are considered “eco-friendly” [21]. In contrast, given the high demand for palm leaves for Domingo de Ramos, it is assumed that their extraction from natural ecosystems inevitably leads to their depletion, even with no knowledge of the ecological and management conditions with which they are often associated [36, 70, 71]. This has generated a prohibition of the use of native palms and the introduction of environmental regulations to limit their use [72, 73]. There is also a tendency for the media in several countries to campaign against the trade in palms [74–76], although studies that demonstrate its possible damaging ecological impacts are rare. However, little or nothing is discussed in the mass media regarding the other side of the coin: the cultural value of *ramos* and the economic income that this festivity provides for thousands of people, which is part of the biocultural heritage of these localities. The case of the Mesoamerican palm *B. dulcis* has played a particularly key role in religious celebrations, as exemplified by the *ramos* made with this species in Nahua communities of Guerrero [59, 77], Hñähñu and mestizo communities of Hidalgo [34–36] and Popolocas from Zapotitlán Salinas, Puebla [61].

The elaboration of *ramos* requires a new leaf with a non-extended (unopened) blade; this leaf is gathered without damaging the trunk or apical meristem of the plant [34]. One or two leaves are gathered from each individual, so the ecological effects of the practice are low, especially considering that *B. dulcis* has the highest leaf production rate of the palms studied in America [36] and the gathering is infrequent. *Brahea* *dulcis* has a great potential for sustainable use because of the gathering strategy employed, and the high productivity and wide distribution of the species [78]. Its role as a key NTFP is prominent due to the extensive traditional knowledge regarding its use and management that is held by the Mesoamerican people [61, 77].

Similarly, the sale of *ramos* represents an important monetary income for thousands of people. For all of the stated reasons, the use of natural populations of *B. dulcis* should be considered even more “eco-friendly” than that of palm leaves obtained from plantations.

The University of Minnesota has a program called “Eco-palms,” directed toward the sustainable gathering of *Chamaedorea* leaves in Mexico and Guatemala for exportation to the USA, generating fairer profits. This initiative has achieved a market of 300 million leaves/year [79]. It is important to consider this initiative that could serve as a reference for the use of *B. dulcis*, which has a high potential for sustainable use, as described above.

This initiative involves the direct use of natural ecosystems, which is relevant to Latin America, where there is a lack of incentives that could be used to demonstrate the true value of nature and invite reflection, rethinking the manner in which conservation has been conceived to date. While observing landscapes that have been plundered and eroded by large-scale mining, agricultural and forestry industries, one cannot continue to conceive that the best form of sustainable use is not to use nature [80], or that environmental protection must sacrifice the cultural aspect, impacting traditional religious symbols [74]. In the words of an interviewee when asked for his opinion on the permits for using the palm: “que si es para privarnos de vender [los ramos] que nos mantengan. No se perjudica a las plantas, pero a veces ellos [el gobierno] hacen cosas y prohíben sin saber” or “if it is to deprive us of selling [the ramos] that support us. The plants are not harmed, but sometimes they [the government] do things and prohibit things without knowing” (Male, 76 years old).

However, Mexican laurel is a threatened species according to NOM-059-SEMARNAT-2010 [45], and thus the ecological effects of gathering must be taken into account. Such use of *Sabal* and *B. dulcis* requires the development of a management plan. According to the interviewees, the reason for regulating use is unknown, since it is not a terminal use, or many people are actually unaware of the need for permits for its use.

### Productive chain and sellers

According to the productive chain and gathering plots for the celebration of Domingo de Ramos, our second hypothesis is rejected. Despite the fact that there are natural populations of *B. dulcis* and *S. mexicana* in our study area, some of the interviewees chose to buy the palm in big markets, mainly in Mexico City, either because they are unaware that there is palm in Hidalgo or because they mistakenly believe that it can be obtained at a better price.

The obtaining chain of the palm is long and includes intermediaries. The most frequent method includes the *ramos* sellers buying the leaves and weaving them. The second most frequent strategy is gatherers weaving and selling the *ramos*. In contrast, in Bolivia, Miguez et al. [8] found that the gatherers rarely also sell the *ramos*.

The sale of *ramo* palms is an occasional and often an annual activity, so the commercial processes can be flexible. For *B. dulcis*, the leaf demand volumes are three orders of magnitude higher for the hat industry in Guerrero than for *ramos* in Hidalgo (Illsley et al. [81] reported 40,000 collectors and this study reports 44). International markets are also reached from Guerrero.

### Ethnobotanical research at regional scale

In general, ethnobiological work is carried out at the local scale, thus obtaining greater detail with which to address specific questions. A challenge in this area of knowledge is to use this local information to analyze issues that go beyond the communities themselves and that can be related to large-scale questions. For example, improving our understanding of why certain plants have been selected for ritual or religious purposes, not being chosen at random, but through complex processes that integrate the empirical with the symbolic and historical [32].

This study, carried out at a regional scale, covering eight geo-culturally distinct regions, documents the symbolic importance of *ramos* palm, a phenomenon that is not unique to any single community and the syncretism of which could be addressed in greater depth using different approaches, such as those anthropology and history.

Likewise, this regional scale allowed us to have an overview of the diversity of plants used and the symbolic similarities in the uses of the *ramos* and to identify a large commercial network that had not been previously identified in the study area and that reflected the complex relationships in NTFP that remain little addressed.

### Palm Sunday in other regions

Palm Sunday is a Catholic celebration with deep roots in Europe. For example, studies on the use of the palm and its relationship with religion in Catalonia mention this unique and characteristic product, especially in the palm grove of Elche, better known as white palm, which is mainly used in the Palm Sunday liturgy [82]. This white palm is divided into two types according to the standard selection based on height and whiteness, leaving the most perfect for “smooth.” These are known as the smooth palms and are intended for men and young people. Another type is the curly palm; the work of curling falls especially on women. Some craftswomen make the ornaments, while others begin to shape the basic structures. One of them, the most skilled, will decide on the design of the most important ones. Afterward, the bouquet is assembled by sewing the ornaments to the structure, which is subject to the curler’s imagination and the palm’s characteristics [82].

The importance of these *ramos* is that every year white palms are sent to the different churches of the province, to the Spanish Royal Family and to the President of the Government, among others. The convent processions throughout Spain are filled with palms, and many of them are exported to different countries to celebrate liturgical acts: England, Portugal, Italy, Belgium, France, Germany and some Scandinavian countries [82].

### Study limitations

In this study, the *rapport* period was minimal. This meant that, despite having the purposes of the study explained to them, in some cases the sellers did not agree to answer the questions for different reasons; distrust, believing that we may have belonged to some government institution; available time, the activities they were carrying out (selling, weaving the *ramos*), the lack of space to talk or even self-doubt, due to which they believed that the questions would be difficult to answer. Given this situation, there is the possibility that the most compromising questions, such as those related to economic income, could have been underestimated. Furthermore, one of the limitations was the number of individuals interviewed for the municipalities studied (essentially 1–9 interviewees per municipality). Another limitation is that the regional scale might not produce sufficient qualitative information on the *emic* perspective of *ramos*. However, the Free List method seems to be a suitable tool for a first approximation since, with the Smith Index, the most frequently cited uses, and those mentioned first, tend to indicate what is uppermost in people's minds and most significant in their lives [83]. In this way, an approach is used concerning what the plant elements used on Domingo de Ramos represent for the people who have dedicated themselves for years to the gathering, weaving and sale of *ramos* and thus allow this tradition to continue to the present day. However, additional efforts and future studies are needed to document the knowledge of the *ramos* in more states or countries. For example: comparative studies with historical data that allow an approximation on the modification of traditional knowledge on the use of *ramos*’ plants; investigations that deeply address the symbolism of plant use, which let us to understand the role plants play not only in religious life but also in shaping identity; commercial aspects for NTFP that rarely addressed in research. These efforts could elucidate a more comprehensive understanding of the biological, social and economic elements, from a comparative perspective.

## Conclusions

This research highlights, from an emic perspective, the contemporary complexity of the cultural significance of the *ramos* and its elements on a regional scale. While the *ramos* represent religious meaning, the principal current uses given to the *ramos* are protection followed by religious. However, it also has uses related to pre-Hispanic beliefs, such as the protection and the usage of some of its elements as medicine. In the same way, the *ramos* elaboration represents a family tradition and a source of extra income for sellers; the main use of the *ramos* is still protection and religious, but contemporary economic, social and cultural aspects are now also part of their value.

Other festivities, overlooked because of their regularity, may in fact have a considerable cultural, biological and economic importance that should also be addressed at different spatial and temporal scales.

## Supplementary Information

**Additional file 1.** The initial interview which was conducted during the week prior to Domingo de Ramos. The interview has 27 questions and was structured 1) for data on the origin and costs of the palms; and 2) if the person collected the palm leaves, detailed questions were asked about the gathering and whether palm weaving is a tradition or if it is practiced solely as a means of generating income.

**Additional file 2. **Interview format which was conducted on Domingo de Ramos. The interview has 65 questions and was structured around: 1) the sociodemographic data of the interviewees; 2) the ramos themselves; and 3) the palms.

**Additional file 3. **Database with information for each ramo: name of the handcrafter, place where the ramo was purchased, its sale price, among others.

**Additional file 4. **Ethnobotanical Collection of ramos, Laboratory of Ethnobiology on Universidad Autónoma del Estado de Hidalgo.

## Data Availability

Databases are available upon request.
