# Supplementary material for: Palm Sunday in central Mexico: among sellers, palms and syncretism
Source: J Ethnobiol Ethnomed. 2023 Jun 3;19:22. doi: 10.1186/s13002-023-00587-3 (PMC10239146; doi:10.1186/s13002-023-00587-3)
Supplement: Supplementary file 1 — Additional file 1. The initial interview which was conducted during the week prior to Domingo de Ramos. The interview has 27 questions and was structured 1) for data on the origin and costs of the palms; and 2) if the person collected the palm leaves, detailed questions were asked about the gathering and whether palm weaving is a tradition or if it is practiced solely as a means of generating income. [file 13002_2023_587_MOESM1_ESM.docx]

Entrevista (semana previa al Domingo de Ramos 2022) **/ Interview (week prior to 2022).**

Número de entrevista **/ Interview number: _________** Localidad **/** **Locality: ______________________________** Fecha **/ Date: ___________** Hora **/ Hour: _________**

| *Nota: las preguntas 1 a 15 son para Todos los participantes (cosechadores o intermediarios). Las preguntas 16 a 27 son únicamente para las personas que cosechan. /* ***Note: questions 1 to 15 are for all participants (gatherers or intermediaries). Questions 16-27 are for gatherers only.*** |
| --- |

*Para TODOS los participantes* ***/ Questions for ALL participants***

1. Nombre del entrevistado / **Name of interviewed:**
2. Edad / **Age:**
3. Lugar de procedencia (dónde vive- municipio y localidad específica) / **Place of origin (where you live- municipality and specific locality):**
4. Lugar donde nació (municipio y localidad específica) / **Place where you were born (municipality and specific locality):**
5. Número telefónico (explicar por qué lo queremos) o en dado caso dirección / **Telephone number (explain why we want it) or, if necessary, address:**
6. Género / **Gender:**

Femenino / **Female:** _________ Masculino / **Male:** _________ Otro / **Other:** _________

1. Escolaridad (grado terminado) / **Education (grade completed):**
2. ¿Cuál es su actividad económica principal? (trabajo principal) / **What is your main economic activity? (main job):**
3. ¿Cuál es su actividad económica secundaria? (otros trabajos) / **What is your secondary economic activity? (other jobs):**
4. ¿Cuántas hojas de palma trajo para vender en ESTE día? / **How many palm leaves did you bring to sell on THIS day?:**
5. ¿En cuánto compró las hojas de palma? Y ¿En cuánto vende las hojas de palma? (Aquí sabremos si solo es intermediario o es cosechador). / **How much did you buy the palm leaves? And how much do you sell palm leaves? (Here we will know if he/she is only an intermediary or a gatherer).**

| Costo de compra**/Purchase cost ($)** | Costo de venta**/Cost of sale ($)** |
| --- | --- |
|  |  |

1. ¿Para qué le es útil el dinero que gana de la venta de palma? / **What is the money you earn from the sale of palm useful for?**
2. ¿Dónde se cortaron las hojas de palma que usted vende en ESTE día? Y ¿Cómo es cada uno de estos lugares? (cerro, cultivo, huerto, otro-cuál, no sé)? / **Where were the palm leaves you sell on THIS day cut? And how is each of these places? (hill, crop, orchard, other-which one, I don't know)?**

| Municipio **/ Municipality** | Localidad(es) específica(s) **/ Specific locality(ies)** | Descripción del lugar (cerro, cultivo, huerto, otro- ¿cuál?, no sé) **/ Description of the place (hill, crop, orchard, other- which one? I don't know)** |
| --- | --- | --- |
|  |  |  |
|  |  |  |
|  |  |  |
| No sabe dónde se cortaron / **Don't know where they cut** |  | |

1. ¿Le da algún tratamiento a las hojas? ¿Cuál? / **Do you give any treatment to the leaves? Which one?**
2. ¿Cuál es el medio de transporte que utiliza para traer las palmas? Y ¿cuál es el *tiempo* *aproximado* que le toma viajar desde su casa al sitio de venta? / **Which type of transportation do you use to bring the palms? And what is the approximate time it takes you to travel from your house to the sales site?**

| Medio de transporte **/ Conveyance** | (Marcar una opción) **/ (Check one)** | Tiempo aproximado **/ Approximate time** |
| --- | --- | --- |
| A pie / **Walking** |  |  |
| En transporte público / **By public transport** |  |  |
| Vehículo particular / **Private vehicle** |  |  |
| Flete / **Freight** |  |  |
| Otro (¿cuál?) / **Other (which one?)** |  |  |

*La siguiente sección es únicamente PARA COSECHADORES* ***/ The following section is for GATHERER only***

1. ¿Con cuántas personas fue usted a cortar la palma que vende en ESTE día? ¿se organizan en grupos? / **With how many people did you go to cut the palm that you sell on THIS day? Are they organized in groups?**
2. ¿Quién le enseñó a cosechar? / **Who taught you to gather?**
3. ¿Esta actividad es una tradición o es ocasional? / **Is this activity a tradition or is it occasional?**
4. ¿Cuánto tiempo le toma cosechar las hojas? / **How long does it take you to gather the leaves?**
5. ¿Qué características deben tener las hojas para ser cosechadas? / **What characteristics must the leaves have to be gathered?**
6. ¿Qué herramienta utiliza para cosechar las hojas? / **What tool do you use to gather the leaves?**
7. ¿Almacena las hojas cosechadas? ¿Por cuánto tiempo? ¿En qué lugar? / **Do you store gathered leaves? How long for? Where in?**
8. Considera que a los artesanos les gustan más las hojas de algún lugar, ¿de cuál(es) y por qué? / **Do you think that handcrafter prefer leaves from somewhere, which one(s) and why?**
9. ¿Tienen en su comunidad algún tipo de normas o acuerdos para regular el uso de la palma para que con el tiempo no se acabe? En caso afirmativo, favor de mencionarlas(os) / **Do you have any rules or agreements in your community to regulate the use of palm in order that it does not end over time? If yes, please mention them.**

|  |
| --- |
|  |
|  |
|  |

1. ¿Alguna vez alguien le ha prohibido vender la palma? En caso afirmativo, ¿me puede platicar qué ocurrió? / **Has anyone ever prohibited you from selling the palm? If yes, could you tell me what happened?**
2. ¿Alguna vez usted o alguien de su comunidad ha intentado obtener los permisos que pide el gobierno para cortar las hojas de palma? SI: _____ NO: _____ ¿Por qué? / **Have you or someone in your community tried to obtain the permits the government requires to cut palm leaves? YES:______ NO:_______ Please explain the reason?**
3. ¿Qué le diría usted al gobierno respecto a los requisitos que éste solicita para la obtención de permisos para aprovechar la palma? / **What would you say to the government regarding its requirements to obtain permits to use the palm?**

*¡Muchas gracias por su tiempo y conocimiento compartido!* ***/ Thank you very much for your time and shared knowledge!***
