# Supplementary material for: Palm Sunday in central Mexico: among sellers, palms and syncretism
Source: J Ethnobiol Ethnomed. 2023 Jun 3;19:22. doi: 10.1186/s13002-023-00587-3 (PMC10239146; doi:10.1186/s13002-023-00587-3)
Supplement: Supplementary file 2 — Additional file 2. Interview format which was conducted on Domingo de Ramos. The interview has 65 questions and was structured around: 1) the sociodemographic data of the interviewees; 2) the ramos themselves; and 3) the palms. [file 13002_2023_587_MOESM2_ESM.docx]

Entrevista (Domingo de Ramos 2022). Número de entrevista **/ Interview (Palm Sunday 2022). Interview number:** ____________________

Localidad **/ Location: ______________** Fecha **/ Date: _______________** Hora **/ Hour: _______**

1. Nombre del entrevistado / **Interviewed name:**
2. Edad / **Age:**
3. Lugar de procedencia (dónde vive- municipio y localidad específica) / **Place of origin (where you live- municipality and specific locality):**
4. Lugar donde nació (municipio y localidad específica) / **Place where you were born (municipality and specific locality):**
5. Teléfono (explicar por qué lo queremos) o en dado caso dirección / **Telephone number (explain why we want it) or, if necessary, address:**
6. Género / **Gender**

Femenino / **Female: ________** Masculino / **Male: ________** Otro / **Other: ___________**

1. Escolaridad (terminada) / **Education (completed):**
2. ¿Cuál es su actividad económica principal? (trabajo principal) / **What is your main economic activity? (main job):**
3. ¿Cuál es su actividad económica secundaria? (otros trabajos) / **What is your secondary economic activity? (other jobs):**
4. ¿Habla usted alguna lengua diferente al español? / **Do you speak any language other than Spanish?** SI / **YES: ______** NO / **NO: _____** ¿Cuáles lenguas habla usted? Nota: especificar que “son válidos” los dialectos / idiomas / lenguas. / **What languages ​​do you speak? Note: specify that the dialects / languages ​​​are "valid".**
5. ¿Se considera usted indígena? / Do you consider yourself indigenous? SI / **YES: __________** NO / **NO: ___________**
6. ¿Es usted jefa o jefe de familia? / **Are you the head of your family?** SI / **YES: __________** NO / **NO: ___________**
7. ¿Cuántas personas conforman su familia? / **How many people make up your family?**
8. ¿Cuántas personas dependen económicamente de usted? / **How many people are financially dependent on you?**
9. ¿Con quiénes vino usted a vender los ramos? (anotar el número en todo lo que corresponda) / **Who did you come with to sell the *ramos*? (write the number in all that apply)**

| Persona / **Person** | Parentesco (pareja, hijos, otros familiares, amigos, compadres) / **Relationship (partner, children, other relatives, friends, co-fathers).** | Género (F, M) / **Gender (F, M)** | Edad / **Age (aprox)*** | ¿Cómo se distribuyen el trabajo? / **How is the work distributed?** |
| --- | --- | --- | --- | --- |
| 1. |  |  |  |  |
| 2. |  |  |  |  |
| 3. |  |  |  |  |
| 4. |  |  |  |  |
| 5. |  |  |  |  |
| 6. |  |  |  |  |
|  |  |  |  |  |
| Total= |  |  |  |  |

*Niño 0-11 años; Adolescente 12 a 17; Joven 18-29 años; Adulto 30-59 años; Adulto mayor de 60 años o más / **Child 0-11 years; Teen 12 to 17; Youth 18-29 years; Adult 30-59 years; Seniors aged 60 or over.**

*Sobre los RAMOS* ***/ About the RAMOS***

1. ¿Cuántos ramos trajo para vender? / **How many *ramos* did you bring to sell?**
2. ¿En cuánto está vendiendo los ramos y por qué? (de qué depende ese costo) / **How much are you selling the *ramos* and why? (what does that cost depend on)**
3. ¿Cómo obtuvo usted estos ramos? Si los compró, ¿cuánto le costaron? / **How did you get these *ramos*? If you bought them, how much did they cost?**

|  | (Marcar todas las posibilidades) **/ (Mark all the possibilities)** | Costo de compra **/ Purchase cost ($)** |
| --- | --- | --- |
| Los elaboro él/ella / **Make them he/she** |  |  |
| Los compró / **You buy them** |  |  |
| Me los dio alguien (familiar o amigo) para que los venda / **Someone (relative or friend) gave them to me to sell** |  |  |

1. ¿Cuánto dinero espera(n) o calcula(n) obtener hoy de la venta de los ramos? / **How much money do you expect or estimate to get today from selling of the *ramos*?**
2. ¿Para qué le es útil el dinero que gana de la venta de estos ramos? / **What is the money you earn from selling these *ramos* helpful to you?**
3. ¿Desde hace cuántos años vende usted ramos en esta fecha? / **How many years have you been selling *ramos* on this date?**
4. ¿Hay algún permiso que tuvo que pagar o conseguir para vender en este Domingo de Ramos? ¿Cuál? / **Is there any permit you had to pay or get to sell on this Palm Sunday? Which one?**
5. ¿Qué plantas o elementos debe de llevar un buen ramo? (lista libre, anotar las respuestas en orden de mención) / **What plants or elements should a good *ramo* have? (free list, write down the answers in order of mention).**

| 1. | 6. | 11. |
| --- | --- | --- |
| 2. | 7. | 12. |
| 3. | 8. | 13. |
| 4. | 9. | 14. |
| 5. | 10. | 15. |

1. Y de los elementos que mencionó, ¿cuáles **no pueden faltar** en el ramo? (anotar las respuestas en **orden de mención**). (¿Qué es INDISPENSABLE que lleve el ramo?) / **And of the elements you mentioned, which ones cannot be missing in the *ramo*? (write the answers in order of mention). (What is INDISPENSABLE for the *ramo* to carry?)**

| 1. | 5. |
| --- | --- |
| 2. | 6. |
| 3. | 7. |
| 4. | 8. |

1. ¿Qué usos tiene este ramo? (lista libre, anotar las respuestas en orden de mención) / **What uses does this *ramo* have? (free list, write down the answers in order of mention).**

| 1. | 6. |
| --- | --- |
| 2. | 7. |
| 3. | 8. |
| 4. | 9. |
| 5. | 10. |

1. ¿Qué simboliza / significa / representa el ramo? (las diferentes palabras son alternativas para preguntar) / **What does the *ramo* symbolize/mean/represent? (the different words are alternatives to ask).**

|  |
| --- |
|  |
|  |
|  |
|  |

1. ¿Qué es una reliquia? (una posible respuesta es “no sé”) / **What is a relic? (a possible answer is "I don't know")**
2. En su opinión, ¿Qué caracteriza a los compradores de estos ramos? / I**n your opinion, what characterizes the customers of these *ramos*?**

| 1. |
| --- |
| 2. |
| 3. |
| 4. |
| 5. |
| 6. |
| 7. |

1. ¿Qué hacen con los ramos que no se venden al final del día? / **What do they do with the *ramos* that don't sell at the end of the day?**______________________________________ _____________________________________________________________________________________________________________________________________________________________________________; habitualmente ¿cuántos ramos le “sobran”? (en % o en número) / **Usually, how many *ramos* do you have "leftover"? (in % or number)** _____________________.

***Sobre las PALMAS / About PALMS***

1. ¿Qué nombres conoce que tiene la palma que se usa para hacer los ramos? (en español u otras lenguas). *Nota:* *observar si es la misma palma o se ve que sean especies distintas (si observa que son diferentes tomar fotografías de ser posible) /* **What names do you know of the palm used to make *ramos*? (in Spanish or other languages). *Note: observe if it is the same palm or if it is seen that they are different species (if you observe that they are different, take pictures if possible).***

| Nombre **/ Name** | Lengua **/ Language** |
| --- | --- |
|  |  |
|  |  |
|  |  |

*Nota: Si se observa que son varias especies, las siguientes preguntas hacerlas sobre la palma que se parezcan más a las fotos enviadas /* ***Note: If it is observed that there are several species, ask the following questions about the palm that most resemble the photos sent.***

1. ¿Cuántas palmas trajo para esta fecha? / **How many palms did you bring for this date?**
2. ¿Cómo consiguió las palmas que utiliza en ESTE Domingo de Ramos? (no en cualquier Domingo de Ramos de otro año) / **How did you get the palms you use on THIS Palm Sunday? (not on any other Palm Sunday of another year).**

| Obtención de hojas de palma **/ Obtaining palm leaves** | (Marcar) **/ Check one** |
| --- | --- |
| Las *cosechó* ella/él misma(o) (*cosechador*) / **Gathered by herself/himself *(gatherer)*** |  |
| Las compró *a un cosechador /*  **He bought them *from a gatherer*** |  |
| Las compró *a un intermediario /* ***Bought them from an intermediary*** |  |
| Las compró, aunque *no sabe* si el vendedor es cosechador o intermediario / **He/she bought them, although he/she *does not know* if the seller is a gatherer or an intermediary** |  |
| Me los dió alguien (familiar o amigo) para que los venda / **Someone (relative or friend) gave them to me to sell** |  |

*Nota: Si es el cosechador ir a pregunta 34. De lo contrario, continúe con la pregunta 33 /*

***Note: If it is the gatherer, go to question 34. Otherwise, continue with question 33.***

1. ¿Cuál fue el acuerdo comercial con la persona que le suministra a usted la palma en ESTE Domingo de Ramos? Y ¿En cuánto compra las hojas? / **What was the trade agreement with the person who supplied you with the palm THIS Palm Sunday? And how much do you buy the leaves?**

| Acuerdo comercial **/ Trade Agreement** | (Marcar una opción) **/ (Check one)** | Costo **/ Cost ($)** |
| --- | --- | --- |
| Encarga la palma con alguien conocido de hace años (red comercial establecida) / **Order the palm with someone you've known for years (established business network)** |  |  |
| Encarga la palma con alguien ocasional /  **Order the palm with someone occasional** |  |  |
| Alguien le da la palma y usted solo le *paga lo que venda /* **Someone gives you the palm, and *you only pay what you sell*** |  |  |
| Alguien le da la palma y se reparten un *porcentaje /* **Someone gives you the palm** **and you share a sale percentage** |  |  |
| No hay un acuerdo fijo. Solo llega a un mercado y compra a partir de la oferta / **There is no fixed agreement. You only arrive at the market and buy from the offer** |  |  |
| Otro (¿Cuál?) / **Other (Which one?):** |  | |

1. ¿Dónde se cortaron las hojas de palma con las que están hechos los ramos para ESTE Domingo? (ya sea que la persona las fue a cortar o bien que se las vendieron)

Y ¿Cómo es cada uno de estos lugares? (cerro, cultivo, huerto, otro-cuál, no sé)? / **Where were the palm leaves with which the *ramos* are made for THIS Sunday cut? (either the person went to cut them or they were sold)**

**And how is each of these places like? (hill, crop, orchard, other-which one, I don't know)?**

| Municipio **/ Municipality** | Localidad(es) específica(s) **/ Specific locality(ies)** | Descripción del lugar (cerro, cultivo, huerto, otro- ¿cuál?, no sé) **/ Description of the place (hill, crop, orchard, other- which one? I don't know)** |
| --- | --- | --- |
|  |  |  |
|  |  |  |
|  |  |  |
| No sabe dónde se cortaron / **Don't know where they cut** |  | |

1. ¿Cuáles usos tiene esta palma?, ya sea que usted los sepa hacer o no.

(Lista libre, anotar las respuestas en orden de mención) / W**hat uses does this palm have? Whether you know how to do it or not. (Free list, write down the answers in order of mention).**

| 1. | 5. |
| --- | --- |
| 2. | 6. |
| 3. | 7. |
| 4. | 8. |

1. ¿Qué usos le da a la palma en su casa o su uso diario? (Lista libre, anotar las respuestas en orden de mención) / **What uses do you give to the palm in your home or its daily use? (Free list, write down the answers in order of mention).**

| 1. | 6. |
| --- | --- |
| 2. | 7. |
| 3. | 8. |
| 4. | 9. |
| 5. | 10. |

1. ¿Conoce alguna historia o leyenda sobre la palma? / **Do you know any history or legend about the palm?**

|  |
| --- |
|  |
|  |
|  |
|  |

Dependiendo de las siguientes respuestas, se le harán unas preguntas y otras no ***/ Depending on the following answers, you will be asked some questions and not others:***

| Señalar las respuestas para decidir cuáles preguntas se harán / **Mark the answers to decide which questions will be asked** | | | Preguntas / **Questions** |
| --- | --- | --- | --- |
| ¿Usted fue a CORTAR la palma? **/ Did you go to CUT the palm?** | SI es  Cosechador / **If you are a**  **Gatherer** | NO es  Cosechador / **If you are not a Gatherer** | **38** a **/ to 53** |
| ¿Usted sabe TEJER la palma? **/ Do you know how to WEAVE the palm?** | SI es  Artesano / **If you are a Handcrafter** | NO es  Artesano / **If you are not a Handcrafter** | **54** a **/ to 65** |
| Si respondió que SÍ a las dos preguntas anteriores se le harán TODAS las preguntas que siguen **/ If you answered YES to the two questions above, you will be asked ALL of the following questions:** | | | **38** a **/ to 65** |

| Si SOLO vende los ramos / **If you ONLY sell the *ramos*** | Hacer preguntas 1 a 37 **/ Ask questions 1 to 37** |
| --- | --- |
| Si VENDE ramos Y TEJE la palma /  **If you SELLS *ramos* AND WEAVE the palm** | Hacer preguntas 1 a 37, y 54 a 65 **/ Ask questions 1 to 37, and 54 to 65** |
| Si VENDE ramos Y CORTA la palma / **If you SELL *ramos* AND CUT the palm** | Hacer preguntas 1 a 53 **/ Ask questions 1 to 53** |
| Si VENDE ramos, CORTA Y TEJE la palma / **If SELL *ramos*, CUT AND WEAVE the palm** | Hacer preguntas 1 a 65 (TODAS) / **Ask questions 1 to 65 (ALL) /** |

*SI CORTA LA PALMA* ***/ IF YOU CUT THE PALM***

1. Aparte del lugar a donde fue a cortar las palmas para esta fecha, ¿a qué otros lugares ha ido a cosechar / cortar la palma en otros años? (anotar los sitios más específicos posibles). Y ¿Cómo son esos lugares (cerro, cultivo, huerto, otro-cuál)? **/ Apart from where you cut the palms for this date, what places have you gone to gather / cut the palms in other years? (write down the most specific sites possible). And what are those places like (hill, farm, orchard, other-what)?**

| Municipio **/ Municipality** | Localidad(es) específica(s) **/ Specific locality(ies)** | Descripción del lugar (cerro, cultivo, huerto, otro-¿cuál?) **/ Description of the place (hill, crop, orchard, other-which one?)** |
| --- | --- | --- |
|  |  |  |
|  |  |  |
|  |  |  |

1. ¿Cuántos días antes del Domingo de Ramos cosecha las hojas? ¿Por qué? / **How many days before Palm Sunday do you gather the leaves? Why?**
2. ¿Le da algún tratamiento a las hojas que cortó para el ramo? ¿Cuál? / **Do you give any treatment to the leaves you cut for the *ramos*? Which one?**
3. En ESTE Domingo de Ramos ¿Con cuántas personas fue usted a cortar la palma? ¿se organizan en grupos? / **On THIS Palm Sunday, with how many people did you go to cut the palm? Are they organized in groups?**
4. En ESTE Domingo de Ramos ¿Cuántas hojas cortaron entre todos los que fueron con usted a cortar las palmas? / **On THIS Palm Sunday, how many leaves did you cut among all those who went with you to cut the palms?**
5. ¿En qué otra época del año cosecha usted hojas de palma? ¿Cuántas llega a cortar? / **What other time of year do you gather palm leaves? How many can you cut?**

| Época / fecha **/ Time / date** | Número de hojas aproximadas que corta **/ Approximate number of leaves you cut** |
| --- | --- |
|  |  |
|  |  |
|  |  |
|  |  |

1. ¿Desde hace cuántos años corta usted hojas de palma? / **How many years have you been cutting palm leaves?**
2. ¿A lo largo de esos años ha observado algún cambio en los palmares donde usted va a cortar? ¿Cómo cuál? / **Throughout those years have you observed any changes in the palm groves where you are going to cut? Like which?**

|  |
| --- |
|  |
|  |
|  |
|  |

1. En su experiencia, ¿el corte de hojas beneficia, perjudica o no afecta a la palma o palmares? ¿por qué? / **In your experience, does cutting the leaves benefit, harm or not affect the palm or palm groves? Why?**

|  | Motivo **/ Reason:** |
| --- | --- |
| Beneficia **/ Benefit** |  |
| Perjudica **/ Harms** |  |
| No afecta **/ Does not affect** |  |
| Otro (cuál) **/ Other which one?** |  |

1. ¿Tienen en su comunidad algún tipo de normas o acuerdos para regular el uso de la palma para que con el tiempo no se acabe? ¿Cómo cuáles? / **Do you have any rules or agreements in your community to regulate the use of palm in order that it does not end over time? Which ones?**

|  |
| --- |
|  |
|  |
|  |
|  |

1. ¿Cuál es el medio de transporte que utiliza para traer las palmas? Y cuál es el *tiempo* *aproximado* que le toma viajar desde su casa al sitio de venta / **What is the means of transportation you use to bring the palms? And what is the approximate time it takes you to travel from your home to the sales site?**

| Medio de transporte **/ Conveyance** | (Marcar una opción) **/ (Check one)** | Tiempo aproximado **/ Approximate time** |
| --- | --- | --- |
| A pie / **Walking** |  |  |
| En transporte público / **By public transport** |  |  |
| Vehículo particular / **Private vehicle** |  |  |
| Flete / **Freight** |  |  |
| Otro (¿cuál?) / **Other (which one?)** |  |  |

1. ¿En qué lugares o plazas NORMALMENTE vende hojas de palma? (anotar los sitios más específicos posibles) / **In which places or squares do you USUALLY sell palm leaves? (write down the most specific sites possible)**

| Municipio **/ Municipality** | Localidad(es) específica(s) **/ Specific locality(ies)** |
| --- | --- |
|  |  |
|  |  |
|  |  |
|  |  |
|  |  |

1. ¿Cuál es el LUGAR MÁS LEJANO de su casa en donde usted ha ido a vender hojas de palma? (AUNQUE SOLO HAYA IDO UNA VEZ) / **Where is the FARTHEST PLACE from your house where you have gone to sell palm leaves? (EVEN IF I ONLY GONE ONCE)**
2. ¿Alguna vez alguien le ha prohibido vender la palma? ¿me puede platicar qué ocurrió? / **Has anyone ever forbidden you to sell the palm? Can you tell me what happened?**
3. ¿Alguna vez usted o alguien de su comunidad ha *intentado obtener los permisos* que pide el gobierno para cortar las hojas de palma? / **Have you or someone in your community tried to obtain the permits the government requires to cut palm leaves?** SI / **YES: _____** NO / **NO: _____** ¿Por qué? **Why?**
4. ¿Qué le diría usted al gobierno respecto a los requisitos que éste solicita para la obtención de permisos para aprovechar la palma? / **What would you say to the government regarding the requirements it requests to obtain permits to use the palm?**

*SI TEJE LA PALMA* ***/ IF YOU WEAVE THE PALM***

1. ¿Qué objetos o cosas sabe elaborar usted con esta palma? (lista libre, anotar las respuestas en orden de mención) / **What objects or things do you know how to make with this palm? (free list, write down the answers in order of mention).**

| 1. | 9. |
| --- | --- |
| 2. | 10. |
| 3. | 11. |
| 4. | 12. |
| 5. | 13. |
| 6. | 14. |
| 7. | 15. |
| 8. | 16. |

1. ¿Cuál es su especialidad en el tejido de palma? / What is your specialty in palm weaving?
2. ¿Usted sabe por qué para el Domingo de Ramos la hoja de palma se utiliza fresca (verde)? / **Do you know why palm leaves are used fresh (green) on Palm Sunday?**
3. ¿Cuáles son las principales razones por las que usted trabaja como artesano? (marcar todas las opciones las que apliquen) / **What are the main reasons why you work as an artisan? (check all options that apply):**

|  | SI / **YES** | NO / **NO** |
| --- | --- | --- |
| La agricultura no le da dinero suficiente /  **Farming doesn't give you enough money** |  |  |
| Para gastos de la escuela / **For school expenses** |  |  |
| Para gasto básico del hogar / **For basic household expenses** |  |  |
| Para emplear el tiempo libre / **To spend free time** |  |  |
| Por mero gusto / **I like the activity** |  |  |
| Por herencia y orgullo familiar / **For heritage and family pride** |  |  |
| Otros (cuáles) / **Others (which ones):** |  |  |

1. Si por alguna razón se agota esta palma, ¿qué otra planta o material usaría para *sustituirla*? ¿cuál prefiere y por qué? / **If this palm runs out for some reason, what other plant or material would you use to replace it? Which one do you prefer and why?**

| Planta o material sustituto / **Substitute plant or material** | ¿Cuál prefiere y por qué? / **Which one do you prefer and why?** |
| --- | --- |
| 1. |  |
| 2. |  |
| 3. |  |
| 4. |  |
| 5. |  |
| 6. |  |
| 7. |  |

1. De acuerdo a su experiencia y conocimiento, ¿en qué mercados los artesanos pueden ir a comprar la hoja de palma? / **According to your experience and knowledge, in which markets can artisans go to buy palm leaves?**

1. ¿Qué lo hace sentir orgulloso de su trabajo con la palma? / **What makes you proud of your work with the palm?**

1. En su opinión, ¿qué le molesta o no le gusta de su trabajo con la palma? / **In your opinion, what bothers you or does not like about your work with the palm?**
2. ¿Ha recibido alguna vez apoyo del gobierno para incentivar su trabajo con la palma? / **Have you ever received support from the governme**nt to encourage your work with the palm? SI / **YES: _____** NO / **NO: ______** ¿Cuál? / **Which one?**

1. En su experiencia, ¿cuáles son las *artesanías de palma* más vendidas? (las sepa o no las sepa hacer- lista libre, anotar las respuestas en orden de mención) / **In your experience, what are the best-selling *palm crafts*? (Whether you know them or not, know how to do them- free list, write down the answers in order of mention).**

| 1. | 6. |
| --- | --- |
| 2. | 7. |
| 3. | 8. |
| 4. | 9. |

1. ¿Cuáles son los lugares turísticos en donde se vende mayormente las artesanías de palma? Especificar localidad y municipio / **What are the tourist places where palm handicrafts are mostly sold? Specify town and municipality.**

| Municipio / **Municipality** | Localidad(es) específica(s) / **Specific locality(ies)** |
| --- | --- |
|  |  |
|  |  |
|  |  |
|  |  |

1. ¿Considera que hay artesanías *que los turistas* prefieren comprar antes que las artesanías de palma? ¿Cuáles? / **Do you think there are handicrafts that tourists prefer to buy over palm handicrafts? Which ones?**

*¡Muchas gracias por su tiempo y conocimiento compartido!* ***/ Thank you very much for your time and shared knowledge!***
