# Supplementary material for: Palm Sunday in central Mexico: among sellers, palms and syncretism
Source: J Ethnobiol Ethnomed. 2023 Jun 3;19:22. doi: 10.1186/s13002-023-00587-3 (PMC10239146; doi:10.1186/s13002-023-00587-3)
Supplement: Supplementary file 4 — Additional file 4. Ethnobotanical Collection of ramos, Laboratory of Ethnobiology on Universidad Autónoma del Estado de Hidalgo. [file 13002_2023_587_MOESM4_ESM.docx]

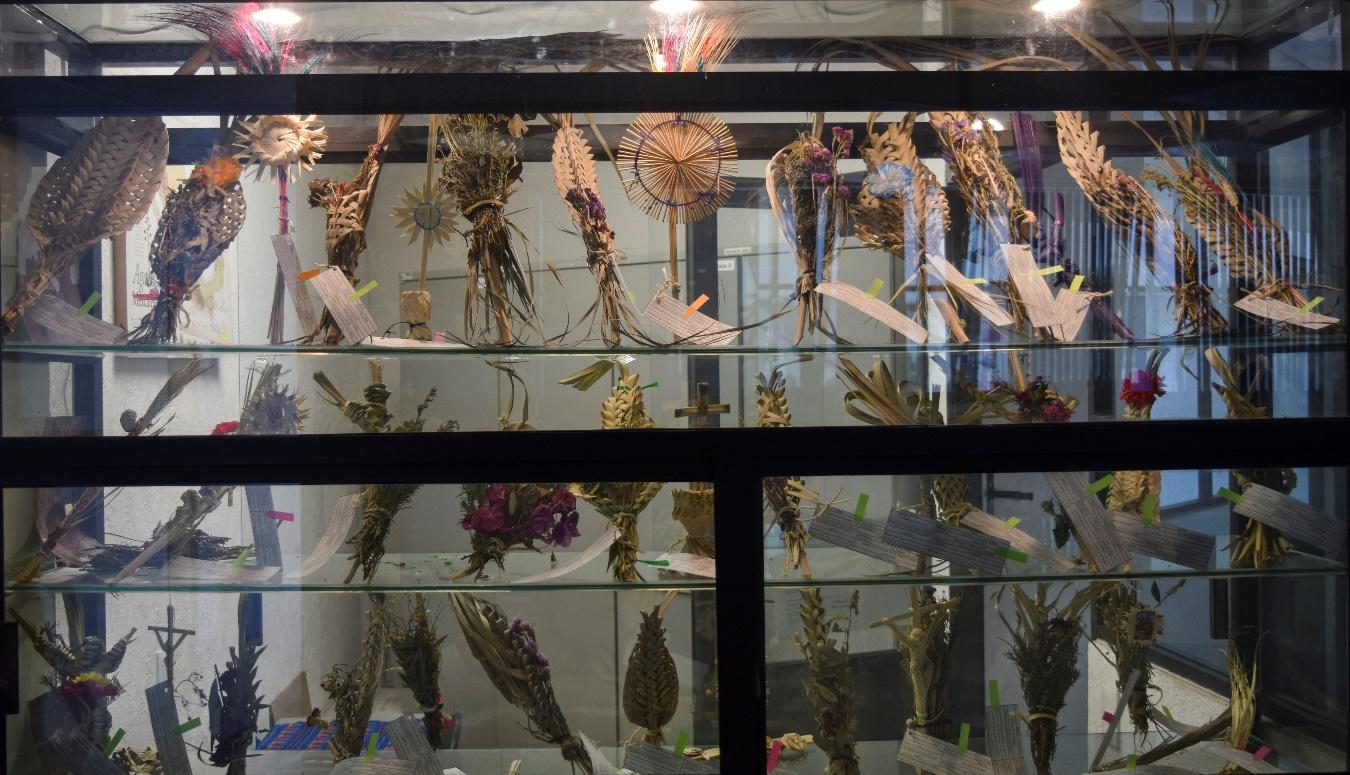

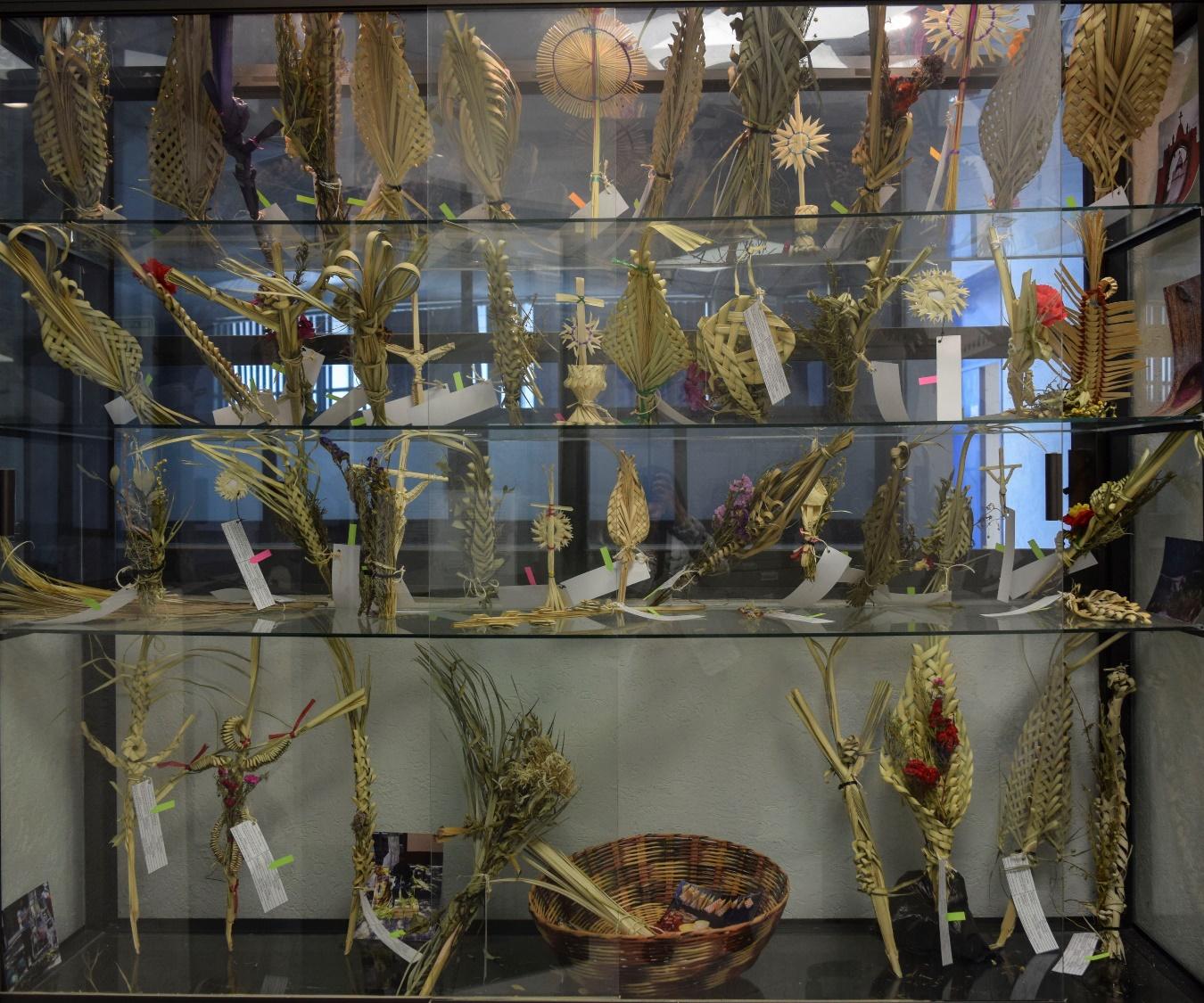


Ethnobotanical Collection of *ramos*, Laboratory of Ethnobiology on Universidad Autónoma del Estado de Hidalgo.
